# Argonaute proteins orchestrate Meiotic Sex Chromosome Inactivation and timing of the spermatogenic transcriptional program

**DOI:** 10.1371/journal.pgen.1012217

**Published:** 2026-06-29

**Authors:** Maria de las Mercedes Carro, Alexis Dziubek, Amanda Touey-May, Elizabeth A. Popkowski, Mark Abdelmassih, Leah E. Simon, Stephanie L. Tanis, Faraz Ahmed, Jennifer K. Grenier, Andrew Grimson, Paula E. Cohen

**Affiliations:** 1 Department of Biomedical and Translational Sciences, Cornell University, Ithaca, New York, United States of America; 2 Department of Molecular Biology and Genetics, Cornell University, Ithaca, New York, United States of America; 3 Biotechnology Resource Center Genomics Facility, Cornell University, Ithaca, New York, United States of America; Dartmouth College Geisel School of Medicine, UNITED STATES OF AMERICA

## Abstract

Argonaute proteins (AGO) are best known for their role in microRNA-mediated post-transcriptional gene silencing. Here, we demonstrate that AGO3 and AGO4, but not AGO2, localize to the sex chromatin of pachytene spermatocytes, where they are required for the transcriptional silencing of XY-linked genes that characterizes Meiotic Sex Chromosome Inactivation (MSCI). Previous findings showed that deletion of *Ago4* (*Ago4*^*-/-*^*)* mildly impairs MSCI and normal spermatozoa production. By contrast, loss of *Ago3* (*Ago3*^*-/-*^*)* does not produce these defects, while combined deletion of *Ago1*, *Ago3*, and *Ago4* (*Ago413*^*-/-*^) leads to severely reduced fertility, accompanied by disrupted autosomal and sex chromosome gene regulation and altered chromatin accessibility in spermatocytes. In *Ago413*^*-/-*^ mice, premature overexpression of spermiogenesis genes during prophase I results in reduced sperm production, abnormal sperm morphology, and impaired fertilization capacity. Together, AGO3 and AGO4 act during prophase I to ensure the timely expression of meiosis-related genes during prophase I while maintaining repression of spermiogenesis-associated genes. These results indicate that AGO3 and AGO4 act in a coordinated fashion in the male germline to orchestrate cell progression in spermatogenesis through temporal regulation of autosomal and sex chromosome genes.

## Author summary

In order to make spermatozoa, germ cells undergo the meiotic specialized cell division, during which the sex chromosomes are temporarily shut down in a process called Meiotic Sex Chromosome Inactivation. In this study, we investigated a subset of the Argonaute family of proteins, best known for controlling gene activity in the cytoplasm. Here we show that two members of this family instead localize and function in the nucleus of germ cells. Using mice lacking these proteins, we found that they help maintain the shutdown of sex chromosomes during meiosis, while also ensuring the timely expression of genes needed for spermatogenesis itself. Here, we provide evidence that these Argonaute proteins also help remodel chromatin in the germ cells, and loss of this regulation causes genes normally needed only later in sperm maturation to switch on too early, leading to fewer sperm, with abnormal morphology and impaired fertility. By studying chromatin structure and gene expression together in the same cells, our work offers a complete temporal picture of Meiotic Sex Chromosome Inactivation, a process still not fully understood in the field.

## Introduction

Mammalian spermatogenesis is a complex developmental program that starts with a diploid precursor spermatogonium and produces four differentiated haploid spermatozoa capable of fertilization. This process involves a series of distinct and stringently regulated developmental programs that must be executed in a precise spatiotemporal manner. First, proliferation and differentiation of spermatogonial stem cells produces B-type spermatogonia, which then further differentiate into spermatocytes. Next, primary spermatocytes enter meiosis, during which they replicate their DNA and undergo two consecutive divisions to form haploid round spermatids. In the third phase, spermiogenesis proceeds as a series of morphological and physiological changes in which round spermatids become elongated spermatids before generating functional spermatozoa [1].

Meiosis encompasses profound changes in gene regulation that accompany the unique and defining nuclear events of this double division cycle. In particular, prophase of the first meiotic division requires a series of nuclear events to achieve accurate homologous chromosome segregation at metaphase I. During leptonema and zygonema of prophase I, homologous chromosomes pair and undergo physical tethering, or synapsis, which is achieved through the assembly of a protein structure called the synaptonemal complex (SC). Concurrent with SC assembly is the recombination process that leads to reciprocal exchange of DNA via crossover formation [2]. Together, SC formation and crossovers serve to maintain homolog interactions through pachynema and diplonema, resulting in the appearance of chiasmata structures by diakinesis [3]. These chiasmata, together with cohesion along the sister chromatids, provide the appropriate tension across the meiotic spindle that ensure accurate chromosome segregation at the first meiotic division.

In male mammals, the lack of homology between sex chromosomes limits synapsis to a small region of homology, referred to as the pseudoautosomal region (PAR) [4]. The remaining portions of the X and Y remain unsynapsed, triggering formation of a heterochromatin-dense domain, the sex body, which restricts transcriptional activity of the sex chromosomes [5,6]. This process, called Meiotic Sex Chromosome Inactivation (MSCI), is initiated by a feed-forward mechanism led by proteins of the DNA Damage Repair pathway (DDR) [7–9]. Initially, DDR proteins are recruited to the sex chromosome axes where histone H2AX is phosphorylated, generating γH2AX [10]. The DDR signal spreads to chromatin loops along with additional repressive chromatin marks, establishing the transcriptionally silenced sex body domain. Following meiosis, XY silencing is partially maintained, characterized by the persistence of repressive histone marks during spermiogenesis [11,12]. The importance of MSCI is highlighted by the existence of checkpoints that cause cell cycle arrest and elimination of germ cells when MSCI defects arise, resulting in infertility [7,13–16]. While the role of DDR accumulation on the sex chromosomes in initiating MSCI is well established, and sex body condensation is a well-characterized downstream consequence of DDR accumulation, the mechanisms underlying sex chromosome-wide gene silencing remain poorly understood. Critical gaps persist regarding the molecular links between DDR signaling and the establishment of chromatin modifications, including the order of histone modifications and their functional interdependencies.

Previous work has shown that the RNA-binding Argonaute (AGO) proteins, AGO3 and AGO4, are localized to the nucleus of pachytene spermatocytes, where they accumulate in the sex body [17]. Four highly conserved mammalian Argonautes exist, AGO1–4, which exhibit diverse expression patterns [18], suggesting a degree of functional specialization. AGOs are best known for their role as partner proteins with microRNAs (miRNAs), mediating post-transcriptional gene silencing in the cytoplasm [19–21]. However, in addition to their participation in post-transcriptional regulation, AGO proteins have been shown to play a role in transcriptional regulation in the nucleus. For example, in *S. pombe*, AGO-dependent small interfering RNA (siRNA) mediated silencing is required for heterochromatin assembly at centromeric repeats [22,23]. Furthermore, in *C. elegans*, Argonaute proteins direct the nuclear RNAi machinery to nascent transcripts to promote co-transcriptional repression and recruit histone modifiers [24–26]. The nuclear role of AGOs in mammalian cells, including in the germline, is less well understood. Recent work has suggested AGO involvement in both enhancing and repressing transcription [27–29], but the underlying mechanisms and the extent to which mammalian AGOs possess nuclear roles has not been established.

All mammalian Argonautes are expressed throughout the somatic and germ cell lineages of the testis, with *Ago2* being highly expressed in Sertoli cells compared to *Ago1*, *Ago3* and *Ago4*. Nuclear localization of AGO2 [30] as well as AGO3 and AGO4 [17] in germ cells implies a role that may be specific to the germline. In line with this, we previously reported that loss of *Ago4* in the mouse leads to upregulation of XY genes and mis localization of DDR factors such as γH2AX and influx of RNA polymerase II (RNAPII) to the sex body, implicating AGO4 in establishment and/or maintenance of MSCI [17]. However, in the absence of AGO4, *Ago3* mRNA and protein are upregulated exclusively in testicular germ cells, along with a concomitant rise in the abundance of AGO3 in the sex body, suggesting that AGO3 can compensate partially for the loss of AGO4 [17].

Here, we have generated two new mouse lines: one a knockout for *Ago3*, and the second harboring a dual MYC-FLAG tag on AGO3. Using these new strains, together with a strain bearing a deletion of *Ago4, Ago1* and *Ago3 (Ago413*^*-/-*^*)*, we have investigated the combined roles of AGO3 and AGO4 in sex chromosome silencing. Importantly, previous data indicate that *Ago1* is not expressed in the germline after the spermatogonial stages [31], allowing us to use these models to study the combined roles of *Ago4* and *Ago3* in spermatogenesis. Using cytogenetic, histological, transcriptomic and proteomic approaches, we show that AGO3 and AGO4 localize to the sex chromosomes of pachytene and diplotene spermatocytes and their dual absence greatly accentuates the MSCI defects observed in single *Ago4* and *Ago3* knockouts. Profiling the interactomes of AGO3 and AGO4, along with simultaneous analysis of differential gene expression and chromatin accessibility in *Ago413*^*-/-*^ germ cells lead us to propose a model whereby AGO3 and AGO4 contribute to transcriptional control during meiosis. Together, our results identify previously unexplored chromatin dynamics of the X and Y chromosomes during meiosis and reveal that loss of AGO proteins leads to alterations in chromatin accessibility in the sex body and autosomes along with changes in transcriptional control during prophase I that are consequential to downstream spermatogenesis. This study advances our understanding of the temporal control of gene expression underlying MSCI and sex chromosome silencing and its relationship with production of healthy spermatozoa.

## Results

### Argonaute protein localization in germ cells

To investigate the role of AGOs in the male germline, we utilized multiple mouse lines, including a previously generated triple knockout mouse strain for *Ago4*, *Ago1* and *Ago3* (Strain # JAX:014152, MGI:101977) and single *Ago3* knockout and *Ago3* tagged mouse lines that we generated. The *Ago413*^*-/-*^ line was created using the MICER system, targeting the three Argonaute genes, which are in tandem in chromosome 4 (S1A Fig), creating a full deletion of *Ago1* and partial deletion of *Ago3* and *Ago4*. Two single *Ago3* knockouts, A1 and A2, were generated using CRISPR-Cas9 gene editing (S1B Fig) and validated using RT-PCR (S1C Fig). Using antibodies specific to each AGO protein, we performed western blot analysis of whole testis lysates and confirmed the loss of AGO1, AGO3 and AGO4 in *Ago413*^*-/-*^ males (S1D-S1F Fig) and loss of AGO3 in *Ago3*^*-/-*^ germ cells (S1G Fig). Since these antibodies were unsuited to immunofluorescence localization in male germ cells, we generated an *Ago3* tagged mouse line by N-terminal insertion of *myc* and *flag* coding sequences into *Ago3* (*Ago3*^myc-flag/myc-flag^). We also utilized an *Ago2* HA epitope-tagged mouse line (*Ago2*^ha/ha^) [28] to characterize its distribution in germ cells. Notably, western blot analysis of germ cells from *Ago3*^myc-flag/myc-flag^ and *Ago2*^*ha/ha*^ males revealed the presence of AGO3 and AGO2 in both nuclear and cytoplasmic fractions (S2A, S2B Fig).

**Fig 1:**
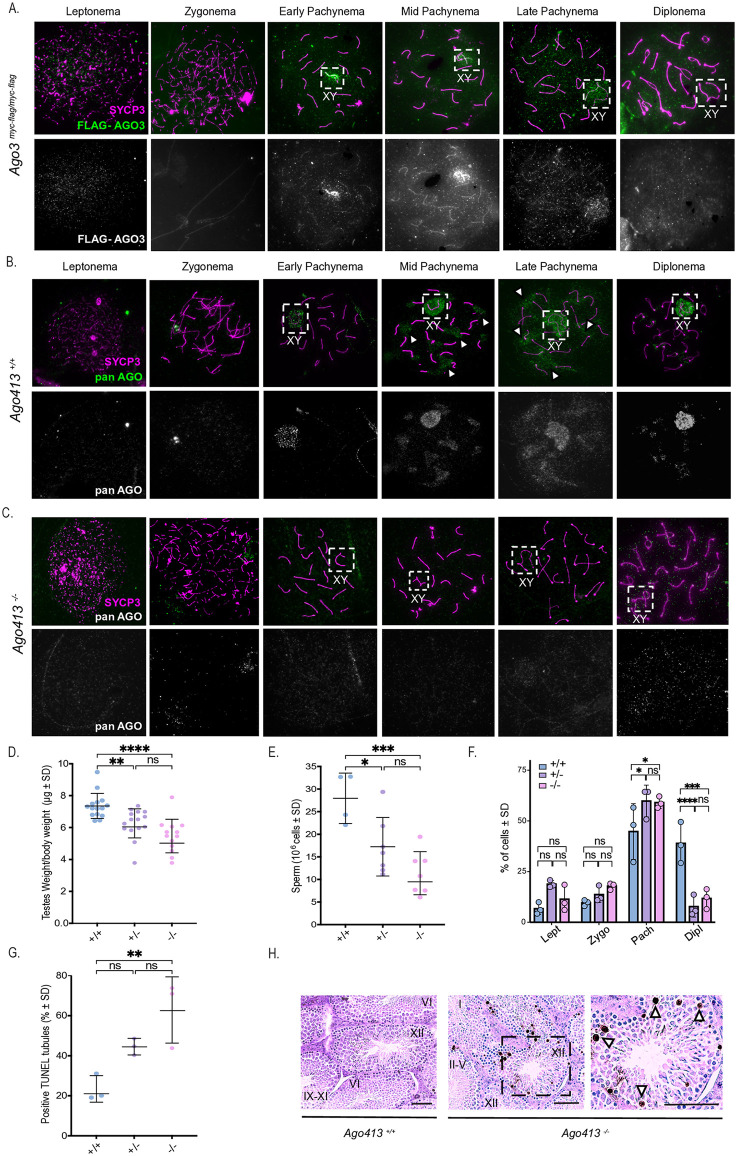
AGO localization during spermatogenesis and *Ago413*^*-/-*^ subfertility phenotype. **(A)** Meiotic spreads of *Ago3*^*myc-flag*^ mice immunostained with anti-SYCP3 and anti-FLAG antibodies, showing AGO3 localization in indicated substages of Prophase **I. (B, C)** Meiotic spreads of *Ago413*^*+/+*^ (B) and *Ago413*^*-/-*^ (C) mice immunostained with anti-SYCP3 and anti-pan AGO antibodies. Dashed boxes indicate the sex chromosomes and white arrows indicate autosomal AGO staining. **(D)** Testis weights relative to body weight for *Ago413*^*+/+*^*, Ago413*^*+/-*^ and *Ago413*^*-/-*^, dots represent data points for individual mice and bars represent the mean ± SD, n = 16, n = 15 and n = 13 respectively. **(E)** Epididymal spermatozoa counts obtained by swim out for *Ago413*^*+/+*^*, Ago413*^*+/-*^ and *Ago413*^*-/-*^, dots represent data points for individual mice and bars represent the mean ± SD, n = 4, n = 7 and n = 7 respectively. **(F)** Percentage of cells in each substage of prophase I obtained after meiotic scoring of prophase I spreads through SYCP3 immunostaining, at least 100 cells per mouse were counted. Bars represent the mean and dots each value ± SD, n = 3. **(G)** Percentage of tubules showing at least one positive TUNEL cell per each genotype. Dots represent individual measurements and bars represent the mean ± SD, n = 3. **(H)** TUNEL staining, bars indicate 40 μm. Insert shows magnification of the *Ago413*^*-/-*^ panel; arrows indicate apoptotic cells. Data were analyzed by ANOVA followed by multiple comparisons (*p < 0.05, **p < 0.005, ***p < 0.001, ****p < 0.0001).

To explore AGO3 localization in the spermatocyte nucleus, we performed prophase I chromosome spreads, probing with an anti-FLAG antibody in *Ago3*^myc-flag/ myc-flag^ males in combination with an anti-SYCP3 antibody to visualize the SC and to define prophase I sub-stages. We observe no AGO3 in the nucleus of leptotene and zygotene spermatocytes (Fig 1A), with chromatin-associated staining of AGO3 becoming apparent in early pachynema, when AGO3 associates with the SCs of autosomes and sex chromosomes. AGO3 localization to the X and Y chromosomes, as assessed by anti-FLAG staining, persists throughout pachynema, spreading through the sex body domain by mid and late pachynema, but is largely absent by diplonema (Fig 1A). In contrast, staining of chromosome spreads from *Ago2*^*ha/ha*^ shows diffuse chromatin associated AGO2 signal (S2C Fig), with predominantly cytoplasmic localization in testis sections (S2D Fig).

To further characterize the localization of all four AGO proteins by immunofluorescence, we used a commercial pan-AGO antibody, which confirms AGO localization in the nucleus of prophase I spermatocytes by early pachynema, with this signal persisting through diplonema (Fig 1B). In addition, pan-AGO staining is present on the pericentromeric heterochromatin in mid-late pachytene to diplotene stage spermatocytes. In spermatocytes from *Ago413*^*-/-*^ males, we observe loss of the XY and pericentromeric heterochromatin pan-AGO signal (Fig 1C). Given that *Ago1* is not detectable in wild-type spermatocytes [31,32], we attribute the heterochromatin signal observed with the pan-AGO antibody to AGO3 and/or AGO4, and neither AGO1 nor AGO2. Taken together, differences in the staining pattern between the anti AGO3 and pan AGO antibodies suggest that AGO3 is removed from the sex body after pachynema, while AGO4 persists until diplonema. Interestingly, AGO3 alone shows nuclear localization in post-meiotic cells, exhibiting a pattern of focal localization near the chromocenter of round spermatids (S2E Fig, asterisks).

### Reduced testis weights and epididymal spermatozoa counts in *Ago413*^-/-^ mice

In mammals, germ cells mature within the testicular seminiferous tubules, that includes germ cells at different stages of development. Contrary to single *Ago4*^*-/-*^ knockouts [17], *Ago3*^*-/-*^ mice show no subfertility phenotype, with testis weights and epididymal sperm counts comparable to their wild-type littermates (S3A, S3B Fig). In contrast, when we compared the reproductive phenotype of *Ago413*^*+/-*^ and *Ago413*^*-/-*^ adult mice to *Ago413*^*+/+*^ males, we observed significantly decreased testis weight (p < 0.01 and p < 0.0001, respectively) and sperm counts (p < 0.05 and p < 0.001, respectively; Fig 1D, 1E). Although loss of *Ago4* alone causes a subfertility phenotype, including reduced testis weights and epididymal sperm counts [17], *Ago413*^*-/-*^ males exhibit a more pronounced phenotype, with a 30% reduction in testis weight (Fig 1D), compared to only a 13% reduction in *Ago4*^-/-^ males [17]. Similarly, sperm counts of *Ago41*3^-/-^ and *Ago41*3^+/-^ males are reduced by 60% and 39% compared to wild-type littermates (Fig 1E), while *Ago4*^-/-^ mice show a more modest 22% reduction [17]. Histological sections of *Ago3*^*-/-*^*, Ago413*^*+/-*^ and *Ago413*^*-/-*^ testes reveal no gross differences in cellular architecture of the seminiferous tubules compared to wild-type males (S3C Fig).

Prophase I progression was monitored using chromosome spreads stained with antibodies against SYCP3. Spreads from *Ago3*^*-/-*^ males do not show differences in prophase I cell population proportions when compared to wild-type males (S3D Fig). However, *Ago413*^*+/-*^ and *Ago413*^*-/-*^ males show a decrease in the proportion of diplotene cells (p < 0.001 and 0.0001, respectively) compared to wild-type littermates, with a concomitant increase in pachytene cells (p < 0.05, Fig 1F). These changes in germ cell proportions indicate that *Ago413*^-/-^ spermatocytes are progressing slower from pachynema into diplonema and/or are dying after pachynema. We confirmed an increase in apoptotic cells in the prophase I to metaphase I layer of the seminiferous tubules of *Ago413*^-/-^ males compared to wild-type by TUNEL analysis (p < 0.01, Fig 1G, 1H).

### Gene dysregulation in germ cells from *Ago413*^*-/-*^ males

**Fig 2:**
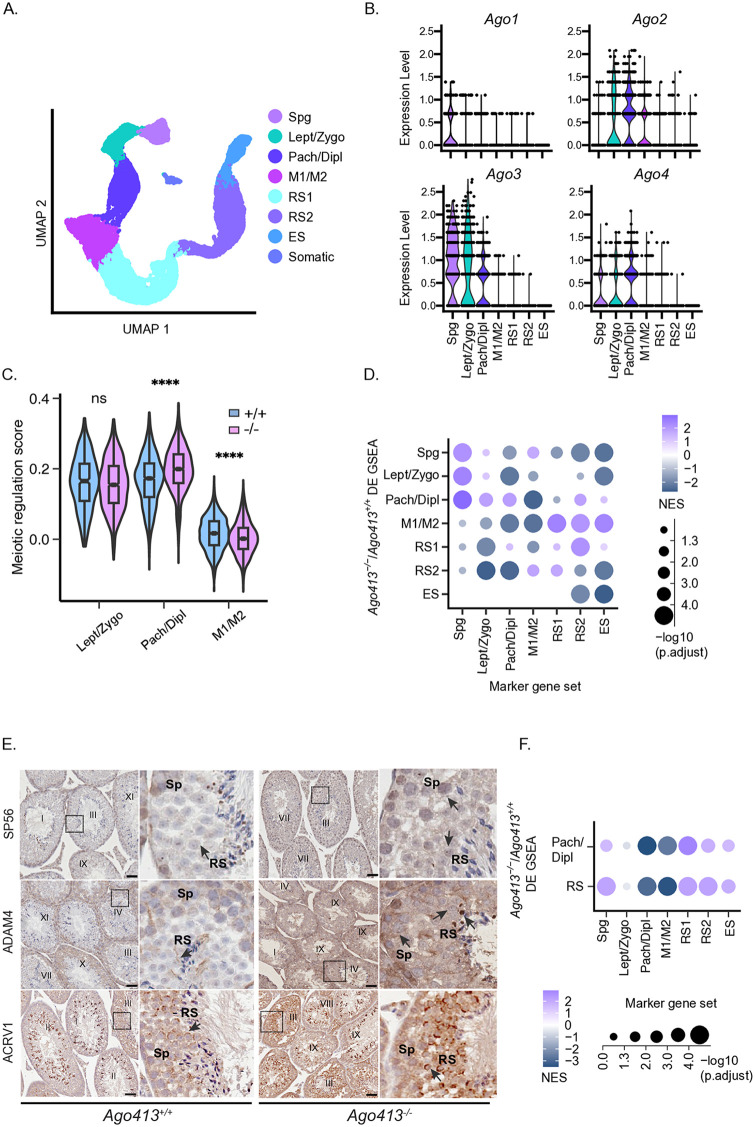
Single Cell RNA-seq analysis of *Ago413*^*+/+*^ and *Ago413*^*-/-*^ germ cells. **(A)** Uniform Manifold Approximation and Projection (UMAP) plot of cell type groupings in scRNA-seq. **(B)** Expression of Argonaute genes in *Ago413*^*+/+*^ germ cell types. **(C)** Violin plot of meiosis regulation gene set scores separated by genotype. Bonferroni-adjusted p-values obtained using Wilcoxon rank-sum test (*p < 0.05, ***p < 0.001, ****p < 0.0001). **(D)** Dot plot showing normalized enrichment score (NES) of cell type marker gene set enrichment analysis. Marker gene sets on X axis and cell clusters on Y axis. Gene sets obtained from wild-type cell marker gene testing (n = 3 for Spg, Lep/Zy, Pachy, Dip/M1/M2, RS1, RS2, and ES respectively). P-values are FDR adjusted and represented by circle size. **(E)** Immunohistochemistry staining of testicular sections from *Ago413*^*+/+*^ and *Ago413*^*-/-*^ males using antibodies against spermiogenesis proteins which genes are upregulated in the scRNA-seq and bulk RNA-seq data in the *Ago413*^*-/-*^ pachytene spermatocyte fraction: *Sp56*, *Adam4*, and *Acrv1*. Bars indicate 40 μm, magnified panel of each image show the spermatocytes (Scyte I) and round spermatid (RS) layers of the seminiferous epithelium where increased IHC signal is observed in *Ago413*^*-/-*^ males. Tubule stages are shown in roman numeral. **(F)** As in (D) but using bulk RNA-seq lof2 fold-change for testing.

Given the pronounced subfertility phenotype observed in *Ago413*^-/-^ mice, we conducted single-cell RNA sequencing (scRNA-seq) of enriched germ cell populations from adult mutant and wild-type testis to assess the impact of combined AGO3 and AGO4 loss on the germline transcriptome (Fig 2A). We classified cell clusters into different germ cell populations based on marker gene expression [33] (S4A Fig). As expected, *Ago1* expression in wild-type males is confined to spermatogonia while *Ago3* and *Ago4* are detected in spermatocytes from leptonema to pachynema (Fig 2B). We then analyzed the expression of genes associated with meiotic progression across different cell clusters to investigate the disrupted progression through meiosis observed in *Ago413*^*-/-*^ males. For this analysis, we generated a meiotic regulation score by comparing gene expression in our data with genes listed in gene ontology terms associated with meiotic cell cycle regulation (see methods for details). We then identified significant differences in this meiotic regulation score in germ cells from *Ago413*^-/-^ males compared to wild-type (Fig 2C). We observed elevated expression of genes governing meiotic progression and checkpoint signaling in pachytene cells, suggesting a shift in the timing or regulation of gene expression required for entry into subsequent meiotic divisions. In contrast, dividing cells in the M1/M2 cluster show a decrease in the expression of these meiotic genes, potentially causing them to move through M phase abnormally. Taken together, these patterns point to a broad disruption in meiotic progression in *Ago413*^*-/-*^ males relative to their wild-type littermates.

To investigate how disrupted meiotic progression in *Ago413*^-/-^ males is related to changes in expression of gene regulatory programs, we identified the signature genes that define each of the wild-type germ cell types by identifying uniquely upregulated genes in each wild-type cluster (S1 Table in S1 Appendix). We then used gene set enrichment analysis (GSEA) to test whether these gene signatures were differentially upregulated or downregulated in *Ago413*^*-/-*^ compared to *Ago413* ^*+/+*^ cells (Fig 2D). We observed that the leptotene/zygotene and pachytene spermatocytes from *Ago413*^*-/-*^ mice have upregulation of spermatogonial gene markers when compared to *Ago413*^*+/+*^ mice. Additionally, pachytene spermatocytes from *Ago413*^*-/-*^ mice also show enrichment of signature genes from earlier leptotene/zygotene clusters (Fig 2D). These observations are consistent with our data indicating slower progression through prophase I (Figs 1F and 2C) and suggest that *Ago413*^*-/-*^ cells are failing to turn off early-stage genes as meiosis advances. Interestingly, we observed the opposite pattern of expression in cells from later stages of prophase I from *Ago413*^*-/-*^ mice. Diplotene spermatocytes and cells in M1 and M2 display premature upregulation of genes that normally become upregulated during spermiogenesis, a later stage in sperm development. This premature upregulation of spermiogenesis genes in the *Ago413*^*-/-*^ germline continues through to early spermatid development, with earlier round spermatids exhibiting upregulation of late round spermatid markers. We sought to confirm whether these changes in mRNA levels impact protein levels in *Ago413*^*-/-*^ germ cells by immunohistochemistry in testis sections for selected spermiogenesis genes: ADAM4, which is involved in sperm migration and binding to the zona pellucida in the oocyte [34], and the acrosomal proteins ACRV1 and SP56 [35,36] (Fig 2E). Increased signal for all these proteins in *Ago413*^*-*/-^ testis sections confirm early upregulation of proteins associated with spermiogenesis in *Ago314*^*-/-*^ germ cells.

We identified biological processes that are associated with genes that were upregulated or downregulated in *Ago413*^*-*/-^ males compared to wild-type males by GSEA (S2 Table S1 Appendix). We observed that the later M1/M2 cluster shows enrichment of gene ontology terms associated with spermiogenesis (S5D Fig), consistent with the early upregulation of round spermatid marker genes in *Ago413*^*-*/-^ males. The round spermatid stages show enrichment of translation and RNA processing terms in upregulated genes (S5E, S5F Fig), which are important for regulation during the round and elongating spermatid stages when transcription is reduced. Moreover, the spermatogonial, leptotene/zygotene, and pachytene clusters of *Ago413*^*-/-*^ males show depletion of spermatid-associated terms, possibly reflecting delayed transcription of genes normally stored for post-meiotic translation (S5A-S5C Fig). Overall, these results indicate that control of the timing of the spermatogenic gene program is altered in *Ago413*^*-/-*^ germ cells.

We validated aberrant gene expression signatures in *Ago413*^*-/-*^ germ cells by bulk RNA seq in sorted pachytene/diplotene spermatocytes and round spermatids using a densitometry BSA gradient [37]. Using our previously identified gene signatures in the scRNA-seq (Fig 2D) we performed GSEA to calculate gene expression differences between Ago*413*^*-/-*^ and *Ago413*^*+/+*^ for both pachytene/diplotene spermatocytes and round spermatids (Fig 2F) and confirmed that both cell populations show premature expression of genes characteristic of later germ cell development stages in *Ago413*^*-/-*^ males.

Taken together, analysis of cell cycle markers indicates that spermatocytes from *Ago413*^-/-^ males show a slower progression during the first stages of prophase I into pachynema, which then transitions to an altered expression profile in dividing spermatocytes in which spermiogenesis genes are mis-expressed. These data, combined with the existence of checkpoints at the pachytene stage that trigger apoptotic pathways in defective cells [38,39], suggest that the increase in cell death detected in the late prophase-to-metaphase layers of the seminiferous tubules of *Ago413*^-/-^ males (Fig 1H) results from cells failing to transition normally from pachynema to division, resulting in their elimination during meiosis I. The lack of TUNEL positive cells in the metaphase and round spermatid layers of the seminiferous tubules of *Ago413*^-/-^ males (Fig 1H) indicates that cells that escape apoptosis at the end of prophase I can progress normally from meiosis I to meiosis II.

### Impaired MSCI in Ago*413*^*-/-*^ mice

**Fig 3:**
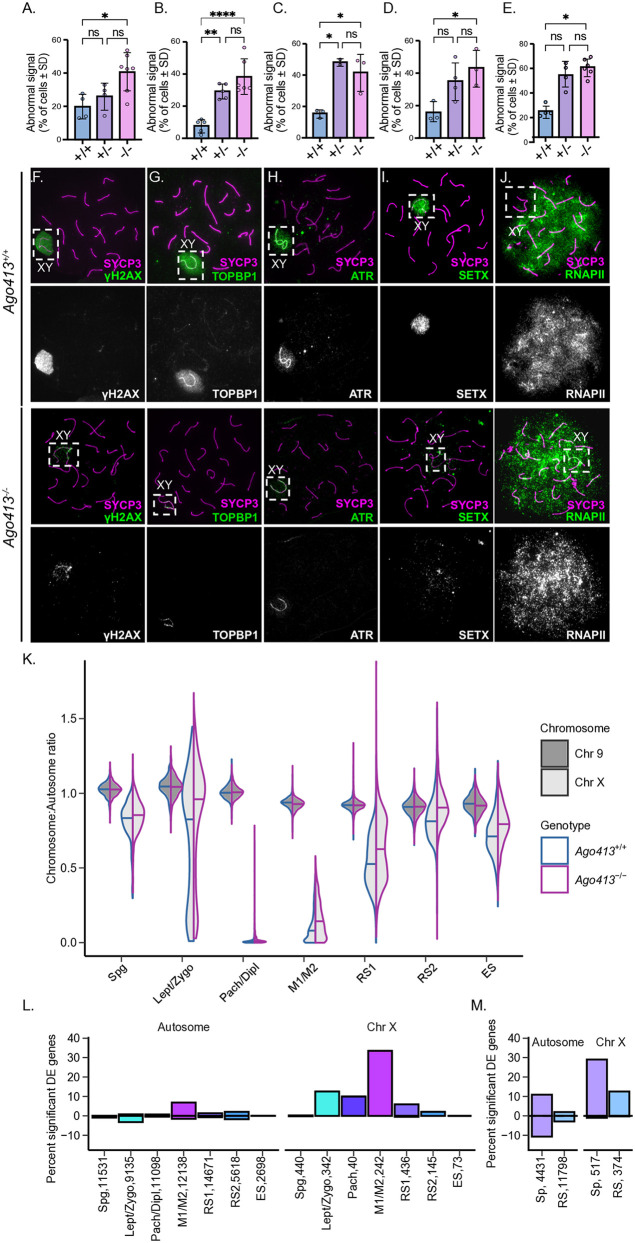
Defective sex chromosome inactivation in *Ago413*^-/-^ germ cells. **(A)** Quantification of aberrant localization for different proteins involved in MSCI, γH2AX, TOPBP1, ATR, SETX and RNA Pol II (order as they appear in the figure) in wild-type, *Ago413*^*+/-*^ and *Ago413*^*-/-*^ pachytene spermatocytes and (B) representative patterns for normal (*Ago413*^*+/+*^) and aberrant (*Ago413*^*-/-*^) localization in chromosomal spreads, dashed boxes indicate the sex chromosomes. Dots represent each replicate and bars represent mean ± SD. Data were analyzed by ANOVA followed by Tukey test for multiple comparisons. (*p < 0.05, **p < 0.01, ****p < 0.0001). **(C)** Violin plots displaying the ratio of the average expression of either X chromosome genes or chromosome 9 genes to the mean expression of all autosome genes at different stages of spermatogenesis. Horizontal bar indicates the median for each cell population. **(D)** Bar plot showing number of genes across cell types called as significantly differentially expressed with adjusted p < 0.05 and absolute value log2FC > 0.5. Percentage above 0 indicates upregulated genes and percentage below 0 indicates downregulated genes. Numbers next to each cell population in the X axes indicate the total number of tested genes. **(E)** As in (D) for bulk RNA-seq. Spg: Spermatogonia, Lep/Zy: leptotene/zygotene, Pachy/Diplo: pachytene/diplotene, M1/M2: metaphase I and II, RS: round spermatids, ES: elongatins spermatids. Scyte I: Spermatocyte, **R.** Sptd: Round spermatid.

To progress to diplonema, spermatocytes must achieve full synapsis along all autosomes and the PAR region of the sex chromosomes by the end of pachynema [13,40,41]. Several lines of evidence motivated us to examine MSCI in *Ago3*^*-/-*^ and *Ago413*^*-/-*^: AGO3 and AGO4 localization to the sex body, alteration in prophase I progression in *Ago413*^*-/-*^ and subtle disruption of MSCI in Ago*4*^*-/-*^ males [17]. Analysis of prophase I spreads from *Ago3*^-/-^ spermatocytes revealed no mis localization of DDR components involved in MSCI initiation and progression (S6A-S6D Fig). In contrast, we found that almost half (40%) of spermatocytes in *Ago413*^-/-^ males exhibit altered localization of γH2AX, TOPBP1 and ATR (Fig 3A, 3B, p < 0.05, p < 0.0001 and p < 0.05 respectively). Similarly, the helicase Senataxin (SETX), a target of TOPBP1 [33] that is required for X and Y chromatin remodeling during meiosis [42,43], also shows reduced localization to the sex body of *Ago413*^*-*/-^ (and *Ago413*^+/-^) spermatocytes compared to *Ago413*^+/+^ cells (48 and 41% respectively, vs 15%, p < 0.05, Fig 3A, 3B). Importantly, these defects in DDR localization to the sex body are not accompanied by crossover or synapsis alternations in *Ago413*^-/-^mice, as evaluated by MLH1 and SYCP1 staining (S6E-S6G Fig). We also analyzed RNAPII exclusion from the sex body in *Ago413*^-/-^ pachytene spermatocytes, as this exclusion is a hallmark event of MSCI. Notably, *Ago413*^-/-^ males show an increase in RNAPII on the X and Y, compared to both *Ago413*^+/+^ males, (Fig 3A, 3B, p < 0.01) and *Ago4*^*-/-*^ males [17] (35% in *Ago413*^-/-^and 30% *Ago41*^*+*/-^vs 18% in *Ago4*^*-/-*^).

To examine MSCI in *Ago413*^*-/-*^ males, we analyzed XY-linked gene expression in our scRNA-seq data. As we previously observed [33], the ratio of XY to autosome gene expression starts decreasing in early prophase I, before pachynema in wild-type cells (Fig 3C). Silencing is completed by pachynema, and this decreased ratio is maintained until the late stages of spermatogenesis (Fig 3D). *Ago413*^*-/-*^ males have an increased number of germ cells with upregulated XY genes (Fig 3C) and substantially more upregulated genes on the X and Y compared to the autosomes for all cell clusters (Fig 3D). We confirmed upregulation of XY transcripts by bulk RNA-seq, using sorted pachytene/diplotene spermatocytes and round spermatids (Fig 3E). This pattern of upregulation manifests from leptotene-zygotene spermatocytes to post-meiotic spermatids, indicating that AGO proteins are needed for preferential silencing of sex chromosomes during both meiosis and spermiogenesis. Analysis of chromosome spreads and single-cell transcriptomics suggests that not all pachytene spermatocytes fail to localize DDR factors to the sex body in *Ago413*^*-/-*^ males. Presumably, the defective cell population is preferentially subjected to apoptosis triggered by the MSCI checkpoint [38,39], while cells without DDR defects are more likely to progress to division and ultimately produce spermatozoa. Together, these results indicate that AGO3 and AGO4 are required for MSCI induction and maintenance.

### Spermatids from *Ago413*^*-/-*^ mice exhibit defective spermiogenesis leading to abnormal spermatozoa function

**Fig 4:**
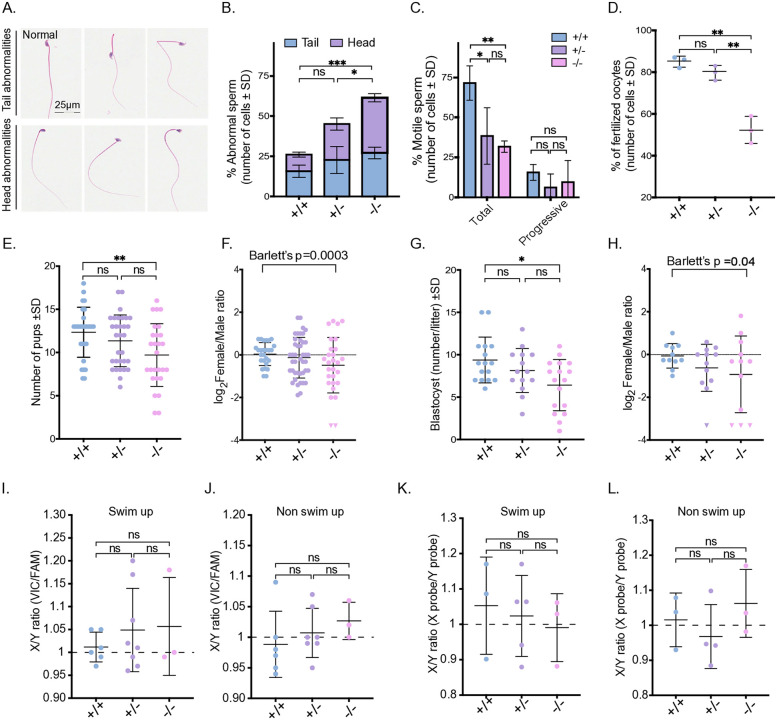
Reproductive phenotype of *Ago413*^*-/-*^ male mice. **(A)** Examples of sperm head and tail morphological abnormalities, normal, bent tail, kinked tail, head tilt, abnormal head shape and lack of acrosome (order as they appear in the figure). **(B)** Percentage of morphological abnormalities for spermatozoa obtained by swim-up for each genotype. Bars represent each mean value ± SD, n = 3. Data were analyzed by Kuskall-Wallis test (*p < 0.05, ***p < 0.001). **(C)** Total and progressive sperm motility obtained by CASA, Bars represent each mean value ± SD, n = 3 (*p < 0.05, **p < 0.01). **(D)** Percentage of fertilized oocytes by IVF using sperm from each genotype. Dots represent each replicate and bars represent mean value ± SD, n = 3. Data were analyzed by Kuskall-Wallis test (*p < 0.05, **p < 0.01). **(E)** Litter size and (F) log2 of female/male pups ratio after natural mating. Dots represent each replicate and bars represent the mean ± SD. Triangles represent pseudo values for female/male = 0. Data were analyzed by ANOVA followed by Tukey test for multiple comparisons (**p < 0.01). Equality of variance of sex ratios data were statistically different according to Barlett’s test (****p < 0.001). **(G)** Number of blastocysts collected at day 3 post-*coitum* and (H) log2 of female/male blastocyst obtained after natural mating as described in panels E and F (*p < 0.05). Equality of variance of sex ratios data were statistically different according to Barlett’s test (*p < 0.05). **(I)** Digital droplet PCR of sperm DNA from different *Ago413* genotypes performed in sperm obtained from swim up motile fraction (I) and non-swim up immotile fraction (J) of epididymal spermatozoa. To target the Y and X chromosome, TaqMan Gene Expression Assay probes against the genes *Sry* with a FAM label and *Rbmx* with a VIC label were used respectively. Dots represent the ratio of VIC to FAM signal for each replicate and bars represent the mean ± SD. Data were analyzed by ANOVA followed by Tukey test for multiple comparisons. **(K)** Sexing of sperm by targeting the X and Y chromosome for Fluorescent in situ hybridization (FISH) in sperm obtained from swim up motile fraction (K) and non-swim up immotile fraction (L) of epididymal spermatozoa Dots represent the ratio of the number of X-fluorescent sperm over Y-fluorescent sperm for each replicate and bars represent the mean ± SD. Data were analyzed by ANOVA followed by Tukey test for multiple comparisons.

Given that a fraction of *Ago413*^*-/-*^ cells evade apoptosis despite MSCI defects, we next evaluated the functionality of the resulting sperm. The morphological and physiological changes that underlie spermiogenic differentiation require gene expression programs that drive acrosome and flagellar formation, nuclear compaction and histone to protamine exchange [44]. We found that sperm morphology is severely impacted in *Ago413*^*-/-*^ males (p < 0.0001), with increased head and tail malformations (Fig 4A, 4B, p < 0.001 for both comparisons). Additionally, *Ago413*^-/-^ sperm exhibit reduced motility (Fig 4C, p < 0.01) and ability to fertilize oocytes *in vitro* (Fig 4D, p < 0.01).

We then asked whether reduced fertilization success of *Ago413*^*-/-*^ males with defective MSCI during meiosis affects preferentially spermatozoa carrying an X or Y chromosome, which would lead to a sex ratio distortion in the progeny. We performed timed mating experiments using wild-type CD1 females, as these produce high numbers of oocytes ovulated per estrous cycle. *Ago413*^*-/-*^ males produce smaller litter sizes (p < 0.01) with a larger variation in the sex ratio of pups compared to wild-type mice (p < 0.001, Fig 4E, 4F). To evaluate the developmental stage at which the decreased litter size and sex ratio variation manifests, we quantified sex ratios in blastocysts. We recovered a lower number of blastocysts from matings between CD1 females and *Ago413*^*-/-*^ males than from matings with wild-type males (Fig 4G, p < 0.01), with increased variance in sex ratios (Fig 4H, p < 0.05), mirroring the weaning data. These results indicate that embryo loss and altered sex ratios occur prior to implantation, rather than during post-implantation development.

To determine if the variation in X and Y bearing sperm from *Ago413*^*-/-*^ males derives from spermatogenic defects, we checked for differences in the proportions of X and Y bearing sperm in the epididymides. Using digital droplet PCR, we observed no significant alterations in X and Y sperm in either *Ago413*^*+/-*^ or *Ago413*^*-/-*^ males, which we verified by DNA FISH (Fig 4I-4L, p > 0.05). These data suggest that the disrupted spermatogenic program in *Ago413*^*-/-*^ males affects sperm carrying each sex chromosome equally and that the sex ratio variance in *Ago413*^*-/-*^ progeny is not due to preferential loss of X or Y-bearing sperm. Taken together, *Ago413*^*-/-*^ animals exhibit reductions in sperm motility parameters and fertilization capabilities concomitant with altered sperm head morphology, accompanied by alterations in the sex ratio of their offspring, arising after sperm production and prior to blastocyst formation.

### *Ago413*^*-/-*^ germline gene expression dysregulation is not explained by canonical miRNA-mediated regulation

To understand the extent to which germline phenotypes in *Ago413*^*-/-*^ males are explained by post-transcriptional dysregulation, we performed small RNA sequencing of enriched pachytene/diplotene spermatocyte and round spermatid cell fractions to determine miRNA expression in the germline and how their expression changes in *Ago413*^-/-^ animals (S7A, S7B Fig; S1 Appendix, Tables C and D). We evaluated miRNAs with high or statistically differential expression to search for evidence of alterations in miRNA-mediated regulation of stringently predicted [45] target genes in the *Ago413*^-/-^ germline. Notably, we observed only small changes between such targets and background genes without target sites in both our scRNA-seq (S7C, S7D Fig) and RNA-seq data (S7E Fig). Moreover, these patterns of mRNA expression do not strongly correlate with changes in miRNA expression. Restricting this testing to just a spermatid development gene set (S8A, S8B Fig) or XY genes (S8C, S8D Fig) revealed similar results, reflecting a minimal alteration in miRNA-mediated post-transcriptional regulation in the *Ago413*^-/-^ germline. An orthogonal approach for detecting miRNA targeting also showed minimal targeting changes in spermatogonia through pachytene spermatocytes (S8C-S8E Fig) and no significant associations after pachynema. Taken together, these data suggest that canonical, cytoplasmic miRNA regulation alone does not explain the disrupted gene regulation we observe in the germline of *Ago413*^*-/-*^ males, an interpretation consistent with the nuclear localization of AGO3 and AGO4.

### AGO4 and AGO3 interact with transcriptional machinery and chromatin remodelers in the male germline

**Fig 5:**
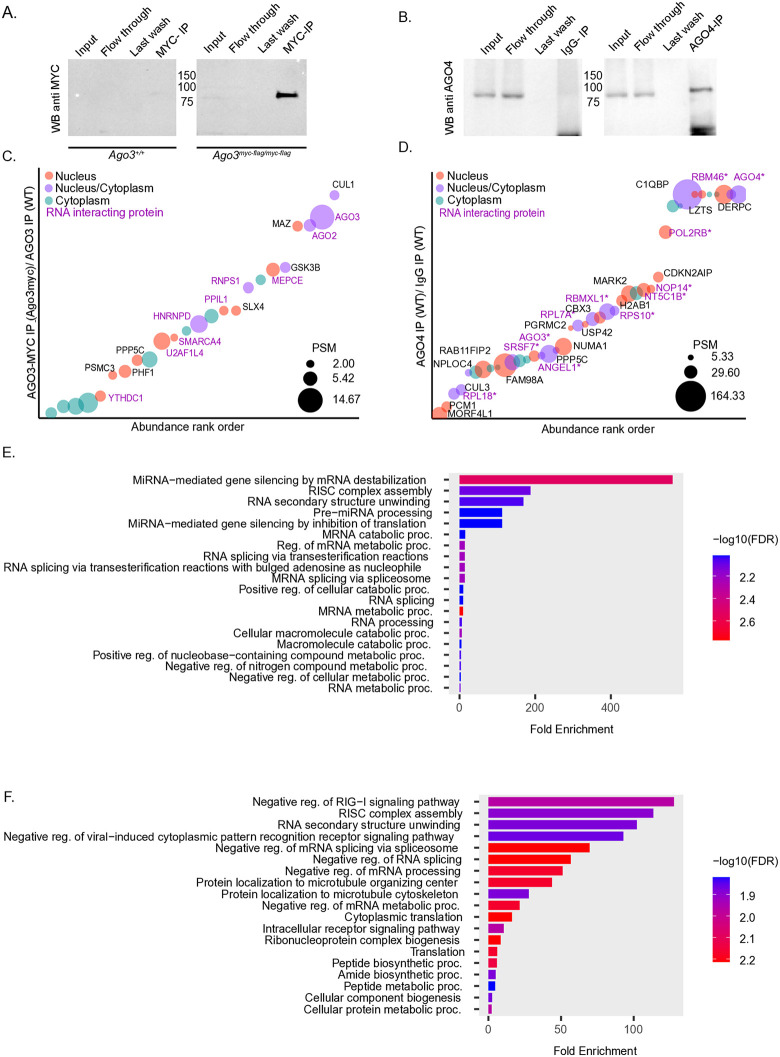
Potential AGO3 and AGO4 interactors in the mouse germline. **(A)** Immunoprecipitation of AGO3 using a MYC antibody in homozygous *Ago3*^*myc-flag*^ germ cells and **(B)** AGO4 using an AGO4 antibody in wild-type germ cells (n = 3). **(C)** Bubble plot showing ranked abundance ratios for candidate protein interactors of AGO3 and **(D)** AGO4. For data visualization, we plot the log_10_-abundance ratios of each interactor against their abundance rank order. Every bubble represents an identified protein, and its size corresponds to the number of peptides found for that given protein. Abundance ratio was calculated as abundance of a given protein in the AGO IP relative to the negative control. **(E)** GO Analysis of candidate AGO3 and AGO4 (F) interactors.

To gain insights into the mechanisms which AGO proteins regulate gene expression in the germ line, we identified AGO3 and AGO4 interacting proteins by immunoprecipitation followed by Mass Spectrometry using antibodies against MYC (for tagged-AGO3) and against native AGO4, respectively (MS, Fig 5). Notably, interacting proteins for both AGO3 and AGO4 are largely predicted to be localized to the nucleus (76% and 72%, respectively; Fig 5C, 5D) and functioning within small RNA pathways and mRNA metabolism (Fig 5E, 5F). Surprisingly, we found that AGO3 and AGO4 have largely non-overlapping candidate nuclear interactors. AGO3-specific interactors include proteins associated with chromatin, including CTCF, the cohesin stabilizer MAZ [46], the H4 interacting protein MEPCE [47], and SMARCA4, the ATPase domain of the chromatin remodeling BAF complex [48]. In contrast, candidate nuclear interacting proteins for AGO4 include a component of RNAPII, POLR2B, as well as YBX2, an RNA-binding protein expressed in pachytene spermatocytes and round spermatids, which binds to promoters in addition to regulating translation [49].. Overall, these findings establish a nuclear role for AGO3 and AGO4 in germ cells and demonstrate that, despite largely non-overlapping interactomes, both argonautes interface with the machinery that regulates the unique transcriptional and chromatin organization of the spermatocyte.

### *Ago413*^*-/-*^ germline shows changes in chromatin accessibility

**Fig 6:**
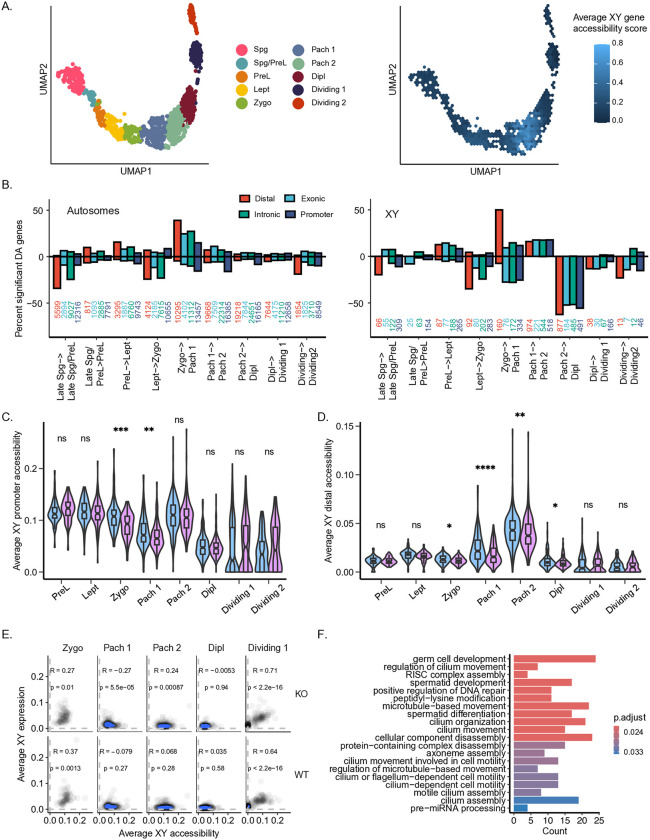
*Ago413*^*-/-*^ mutant cells have decreased XY accessibility. **(A)** UMAP hex plot of mean XY gene accessibility score of wild-type cells. **(B)** Bar plot of percentage of peaks tested for differential accessibility between later and earlier cell stages that are significant with p-value < 0.05. Peaks are separated into categories based on position within genome. Negative and positive values indicate decreased and increased accessibility respectively. **(C-D)** Violin plot and bar plot displaying the average accessibility of XY promoter (C) and distal (D) peaks at different stages of spermatogenesis separated by genotype. P-values calculated with two-sided Wilcoxon rank sum test (*** p < 0.001, ** p < 0.01, * p < 0.01). **(E)** Density and scatter plot of XY promoter accessibility versus expression, R: Pearsons’s correlation coefficient. **(F)** Bar plot of enrichment analysis of significantly downregulated peaks in diplotene cells.

IP-MS data of AGO3 and AGO4 interactors indicate that altered chromatin regulation may contribute to the transcriptional changes and reproductive phenotype seen in *Ago413*^*-/-*^ spermatocytes. To investigate how chromatin and transcriptional regulation are altered in *Ago413* ^*-/-*^ males, we used Multiome scATAC-seq (single cell assay for transposase-accessible chromatin sequencing, paired with single nucleus RNA-seq) and analyzed changes in chromatin accessibility in the germline of *Ago413*^*-/*^ males. Since accessibility dynamics on the X and Y chromosomes during MSCI had not previously been studied at a single cell level, we first investigated baseline accessibility in wild-type mice. After clustering our cells (S9A Fig), we determined the mean gene accessibility score on the X and Y chromosomes (Fig 6A) and autosomes (S9B Fig) per cell. We observed that XY gene accessibility remains relatively consistent across late spermatogonia and early spermatids. Strikingly, the XY chromatin accessibility of spermatocytes increases in the later pachytene cell cluster (Fig 6A), a time at which transcriptional silencing occurs. When testing differential expression of individual peaks across stages for the sex chromosomes (Fig 6B), we found that XY peaks become less accessible from leptotene to zygotene, as expected given the initiation of MSCI at this stage. However, XY peaks distal to genes then become more accessible at the zygotene to pachytene transition (Fig 6B), and both genic and distal peaks increase in accessibility as pachynema progresses, before becoming less accessible in the diplonema and dividing cells clusters (Fig 6A, 6B). This pattern contrasts with the autosomes, where peaks are more accessible throughout pachynema compared to earlier stages (Fig 6B), consistent with the characteristic burst in transcription on the autosomes during this stage [50].

After establishing the chromatin dynamics of the wild-type germline, we compared *Ago413*^*-/-*^ and wild-type gene expression from the multiomic analysis. Nuclear XY transcript levels are significantly increased in *Ago413*^*-/-*^ spermatocytes (S10A Fig), consistent with the upregulation we saw in scRNA-seq data (Fig 3D). As nuclear RNA more directly reflects transcriptional activity, these results suggest that loss of nuclear AGO localization primarily affects transcriptional regulation rather than post-transcriptional processes. Importantly, differential expression analysis of unspliced XY reads, an effective proxy for transcriptional output [51], in *Ago413*^*-/-*^ cells compared to wild-type reveals a greater percentage of significantly differentially expressed genes than when using spliced reads from the same data (S10B Fig). This analysis further suggests that the changes in nuclear RNA levels in *Ago413*^*-/-*^ cells derive from changes in transcription. We next investigated how chromatin accessibility is impacted in the *Ago413*^*-/-*^ spermatocytes compared to those of wild-type littermates. Surprisingly, the upregulation of gene expression we observe in XY chromosomes in *Ago413*^*-/-*^ males is not associated with an increase in chromatin accessibility. Both promoter (Fig 6C) and distal regulatory elements (Fig 6D) in the sex chromosomes show reduced mean accessibility during pachynema in *Ago413*^*-/-*^ spermatocytes. Thus, the observed gene upregulation is not driven by increased chromatin accessibility, but rather likely reflects disruption of the sex chromosome–specific chromatin organization established during MSCI. Consistent with this, in *Ago413*^*-/-*^ early pachytene cells, XY gene expression is significantly negatively correlated with chromatin accessibility (Fig 6E). To determine the relative timing of changes in gene expression and chromatin accessibility in *Ago413*^*-/-*^ germ cells, we plotted the average of accessibility and gene expression across the differentiation trajectory as measured by pseudo time (S12 Fig). This analysis shows that XY accessibility at both promoter and distal peaks is decreased in the *Ago413*^*-/-*^ germ cells by leptonema (S12 Fig). In contrast, the upregulation of gene expression observed in these cells is only distinguishable later in pachynema, indicating that this change in transcription is likely a downstream consequence of accessibility changes. Importantly, these changes are not indicative of a global reduction in accessibility across all chromatin. Autosomes in *Ago413*^*-/-*^ germ cells display regulatory element specific alterations: promoter regions become more accessible across all germ cell stages, whereas distal elements become less accessible, with the exception of pre-leptotene and zygotene stages (S11A, S11B Fig). Interestingly, we found that regions of the genome with lower chromatin accessibility in *Ago413*^*-/-*^ diplotene spermatocytes are enriched for spermiogenesis genes (Fig 6F), consistent with our previous observation that these genes are prematurely expressed in spermatocytes and round spermatids from *Ago413*^*-/-*^ mice. Taken together, these data identify transient alterations in XY chromatin accessibility states during MSCI that may be essential for the proper timing of subsequent gene expression programs, with AGO3 and AGO4 proteins playing a role in this regulatory priming.

## Discussion

### Defining distinct, yet overlapping nuclear roles for AGO3 and AGO4 in mammalian meiosis

The Argonaute proteins are essential for miRNA-mediated post-transcriptional gene regulation across multiple cell types and species [21]. The extent to which mammalian AGOs function in the nucleus, and/or independently of miRNAs, to impact transcription and transcription-related events has remained elusive, despite extensive studies in *S. pombe* and *C. elegans* indicating roles for AGOs in co-transcriptional repression and recruitment of repressive histone marks [22–26]. We previously showed that AGO3 and AGO4 localize almost exclusively to the heterochromatin-rich XY-containing sex body of mouse spermatocytes [17]. Extending these findings here, we reveal distinct localization patterns for AGO2, AGO3 and AGO4 during spermatogenesis. In spermatocytes, AGO2 has diffuse nuclear localization but remains enriched in the cytoplasm, while AGO3 and AGO4 have a specific nuclear localization. AGO3 localizes to the SCs of all chromosomes in early pachynema and becomes restricted to the sex body by late pachynema. AGO4 localizes to the sex body from early pachynema and persists until diplonema. Additionally, we observe AGO localization to pericentromeric heterochromatin of all chromosomes in pachytene and diplotene spermatocytes, which we attribute to AGO4 as neither AGO2 nor AGO3 show such localization.

The reproductive defects described here for *Ago413*^*-/-*^ mice show that AGO3 and AGO4 regulate the timing of gene expression during spermatogenesis and participate in MSCI. Four lines of evidence indicate that these functions of AGO3 and AGO4 are driven by events in the nucleus. First, the enrichment of AGO3 and AGO4 in the nucleus of prophase I cells. Second, candidate interacting proteins of AGO3 and AGO4 function in nuclear processes such as transcriptional regulation and chromatin remodeling. Third, de-repressed spermiogenesis genes and XY-linked genes are not enriched in miRNA targets of differentially expressed miRNAs in *Ago413*^*-/-*^ males, and XY-linked genes are detectably upregulated when tested using unspliced reads as a proxy for transcription. And fourth, these upregulated genes are associated with altered chromatin accessibility.

Nuclear roles for the RNAi machinery are well established in organisms such as *C. elegans, S. pombe* and *A. thaliana*, where argonaute proteins regulate chromatin dynamics [22–26,52–54]. In mammals, despite many reports showing their nuclear localization [27,30,55–57], the extent and mechanisms of nuclear argonaute function remain poorly understood. One model proposes that post-translational modifications and changes in protein interactions between AGO proteins and other RNA binding proteins modulates their translocation to the nucleus in a context dependent manner [58–60]. Beyond small RNA-mediated mechanisms similar to those in *C. elegans, S. pombe* and *A. thaliana*, AGOs within the sex body may also promote XY silencing through a miRNA-independent mechanism mediated by specific nuclear protein and/or RNA interactions. The presence of specific protein interactors in the sex body and other heterochromatin domains in the nucleus such as pericentric heterochromatin might determine both the localization of AGO proteins to these regions in the germ cell and the unique functions of AGO3 and AGO4 at these locations. The limited shared nuclear interacting partners suggest that AGO3 and AGO4 act through partially distinct but complementary mechanisms to achieve meiotic silencing. Consistent with this context-dependent specialization, we observed that single *Ago4* knockout mice display a mild fertility phenotype [17], while single *Ago3* knockout mice do not exhibit overt reproductive defects, with normal meiotic progression and proper localization of sex body markers in pachytene spermatocytes. In contrast, loss of both AGO3 and AGO4 resulted in a substantially more pronounced phenotype than single knockouts, indicating that AGO3 and AGO4 have partially redundant roles during spermatogenesis, whereby AGO4 can compensate for the loss of AGO3 under normal conditions but AGO3 becomes consequential when AGO4 function is lost.

### Gene transcription and chromatin accessibility are decoupled in male mouse prophase I

Our single-nucleus ATAC-seq data show that XY chromatin accessibility is highly dynamic throughout prophase I. Early pachynema is characterized by broad chromatin compaction across gene bodies in the XY chromosomes but increased accessibility at distal gene regions. By late pachynema the sex chromatin experiences a transient increase in accessibility across gene bodies followed by near-complete closure by diplotene. Importantly, these accessibility dynamics are not tightly linked to transcriptional output, as both scRNA seq and single nucleus RNA seq data show that MSCI is initiated by zygonema and persists post-meiotically, indicating that 1) MSCI precedes full sex body formation and 2) chromatin accessibility and transcriptional silencing can be partially uncoupled in pachytene spermatocytes. Open chromatin can be transcriptionally inactive, while many actively expressed genes exist within compact chromatin environments, reinforcing the view that transcriptional activity does not always follow a simple open versus compact chromatin model [61–64]. Our observations of XY chromatin dynamics in wild-type spermatocytes suggest that the XY chromatin undergoes a transient increase in accessibility in late pachynema, concordant with previous observations that pachytene spermatocytes show higher XY accessibility than differentiating spermatogonia at intergenic and intronic regions [65]. This transient accessibility is lost in diplonema, when the sex body switches to a DAPI-dense heterochromatin compartment [66]. In *Ago413*^*-/-*^ spermatocytes, reduced accessibility is associated with an increase in XY transcription. These data suggest that transient and localized increased chromatin accessibility might be needed for recruitment of the transcription silencing machinery at regulatory regions, and this is important for MSCI maintenance after DDR signaling pathways. Interestingly, this requirement for regulatory accessible chromatin before chromosome wide transcriptional inactivation has also been shown during somatic X chromosome inactivation, along with evidence for transcription downregulation happening before chromatin compaction [67–69].

It has been postulated that chromatin domains can behave like liquid condensates. Thus, physical behavior of the chromatin varies at different scales and establishes DNA accessibility and transcriptional regulation at the local level [70–73]. Notably, recent work has discovered that MAPS (Male Pachynema Specific Protein) is essential for sex body formation due to phase separation properties [74]. Like *Ago413*^*-/-*^, *Maps*^*-/-*^ spermatocytes show increased XY expression coupled with decreased chromatin accessibility, predominantly at the distal regions of genes. Together, these observations support the idea that the sex body acts as a phase-separated nuclear compartment [75–77,78,79] that undergoes stage-specific remodeling and requires accessibility at regulatory regions for efficient recruitment of MSCI machinery. Such a model would indicate that the ATAC-seq signal on the X and Y (in pachynema) is a composite of conventional chromatin accessibility overlayed with the impact of phase-separation. The reduced spreading of DDR factors along chromatin loops of the XY in *Ago413*^*-/-*^ spermatocytes by mid pachytene indicates that AGO3 and AGO4 play a role in promoting the chromatin landscape within the phase-separated sex body that facilitates DDR factors recruitment to regulatory regions to the XY chromosomes.

### A nuclear role for argonaute proteins in orchestrating spermatogenesis

Loss of AGO3 and AGO4 disrupts gene expression timing during prophase I, delaying the expression of genes required for early meiotic progression while causing premature expression of genes needed in later stages. Taken together, our findings position AGO3 and AGO4 as upstream nuclear coordinators of the spermatogenic transcriptional program, linking the specialized demands of sex chromosome silencing with broader temporal control of gene expression across meiosis and spermiogenesis. We postulate that AGO3 and AGO4 in the sex body promote the chromatin environment required for ongoing MSCI and for the persistence of sex chromosome repression into the post-meiotic phase, thereby helping to preserve the epigenetic memory of meiotic silencing through spermatid differentiation. At the same time, the early association of AGO3 with autosomal synaptonemal complexes, together with the widespread mistiming of meiotic and spermiogenic gene programs in germ cells from *Ago413*^-/-^ males, suggests that AGO3 and AGO4 functions are not limited to the sex chromosomes but may extend to autosomal regulatory networks that govern orderly spermatogenic progression. Although some of the gene dysregulation we observe in *Ago413*^*-/-*^ germ cells likely derive from alterations in miRNA-mediated posttranscriptional control, two observations suggest that the autosomal aberrant gene expression we observe may also derive from chromatin-associated roles for AGO3 and AGO4. First, the gene upregulation observed in *Ago413*^*-/-*^ germ cells is poorly explained by changes in canonical miRNA regulation, and second, the AGO3 and AGO4 protein interactomes point strongly to non-canonical functions in the germline nucleus, linking these proteins to chromatin remodeling, transcriptional regulation, RNA metabolism, and nuclear architecture. We therefore propose that AGO3 and AGO4 act through distinct but coordinated nuclear mechanisms to organize chromatin state, reinforce sex body-specific repression, and prevent premature activation of the spermiogenic program during prophase I. In this model, loss of AGO3/AGO4 disrupts not a single pathway but the timing logic of spermatogenesis itself, producing a cascade of meiotic, post-meiotic, and functional sperm defects. These studies reveal AGO3 and AGO4 as unexpected regulators of mammalian germline development and identify Argonaute-dependent nuclear regulation as a previously underappreciated mechanism connecting MSCI, chromatin organization, and production of functional spermatozoa.

## Methods

### Ethics statement

All mice used in this work were handled following institutional guidelines under the protocol 2013–0041, approved by the Institutional Animal Care and Use Committee (IACUC) at Cornell University.

### Experimental animals

All mouse strains were backcrossed at least five generations and maintained on a C57BL/6J background (Jackson Laboratory). Mice were maintained under strictly controlled conditions of constant temperature, 12-hour light/dark cycles, and provided food and water *ad libitum*. All mice in this study were euthanized between 8 and 18 weeks by CO_2_ asphyxiation. Genotyping was conducted using ear snips obtained from mice at 4–6 weeks of age, followed by DNA extraction and PCR using the primers indicated on the resource table for each mouse line. The *Ago413* mouse line, strain #:014152, was obtained from Jackson Laboratory and maintained in our mice facilities by +/- by +/- crosses. This line was originally made in the 129 mouse strain and backcrossed into B6/C57 mice at least 4 times by the Hannon Lab, the donor lab. The line was created using the MICER system. Cell lines derived from embryonic stem cells- AB2.2 129S7/SvEvBrd-*Hprt1*^+^- were transfected with 5’Hprt and 3’Hprt vectors containing exons from either side of the targeted region within exon 6 of *Ago3* and exon 15 of *Ago4*, single loxP sites and a neomycin or puromycin drug selection cassette. Resultant mice were crossed with a Sox2-cre deleter mouse on a mixed C57BL/6 and Swiss Webster background to delete the floxed region, removing full *Ago1* and partial *Ago3* and *Ago4* genes constitutively. Triple knockouts produce mRNA from *Ago3* and *Ago4* genes with a premature stop codon located just before the *Ago4* chimeric breakpoint and several premature stop codons just before and after the *Ago3* chimeric break point. Elimination of expression of all 3 Argonaute genes was validated by the Hannon Lab by quantitative RT-PCR of embryonic day 13.5 (E13.5) mouse embryonic fibroblasts. Here, we confirmed this by analysis of our germ cell scRNA-seq data, and Western Blot of protein lysates from testis of *Ago413*^*-/-*^*, Ago413*^*+/-*^ *and Ago413* ^*+/+*^ mice (S1 Fig). The *Ago3*^*-/-*^ lines were generated in the Cohen Lab by CRISPR/Cas9 knock out of the *Ago3* gene. Two guide RNAs (5’- CTGATATACAGCTCATAAAT -3’ and 5’- GACGGCGACAATCCCGAGCG -3’) targeting chromosome 4 were used to delete the entire coding sequence of the *Ago3* gene. Two founders were identified by PCR screening carrying the deletion, founder A1 and A2, resulting in full deletion from position Chr4: 126220355–126323850 in founder A1 and from Chr4: 126220238–126323532 in founder A2. The deletion was validated by PCR from cDNA using four sets of primers targeting different regions of the cDNA (see primer sequences in S1 Resources) and by western blot using protein lysates from germ cells and antibodies against AGO3 (S1 Fig).

The *Ago3*^*myc-flag*^ line was generated in the Cohen Lab by CRISPR/Cas9 knock in of the *myc/flag* coding sequence 41 base pairs upstream from the start codon, which corresponds to the N-terminal domain of the AGO3 protein. The single-stranded oligodeoxynucleotide used was the following: CGCTCCTCGCCTCTGTGGTGGCACCCTTCTCTCGTGAAGCACTCCCC (homology arm) CCA (PAM) GCTCCATGA (guide) ATG (start site) GAACAAAAACTTATTTCTGAAGAAGATCTG (myc) GACTACAAAGACGATGACGACAAG (flag) ATG (start site) GAAATCGG (guide) CTCCGCAGGTGAGGCGAGCTGCGGGACAGGGCAGGTGGG (homology arm).

The epitope tag was validated by Western blot, using protein lysates from whole testis and germ cells in *Ago3*^*my-flag/myc-flag*^ and littermates *Ago3*^*+/+*^ and an anti MYC tag antibody, where only *Ago3*^*myc-flag/myc-flag*^ lysate showed a band running at ~100 kDa (S2 Fig).

### Immunoblotting

Protein extraction from whole testis or single germ cell suspensions was performed by lysing the cells in ice cold buffer (0.5M Tris-HCl pH 8.0; 1% NP-40; 150mM NaCl; 5mM EDTA plus protease inhibitors, Roche complete® tablet) followed by a sonication step (23 amplitude, 0.4 seconds on, 0.2 seconds off, 12 seconds total). Then, the lysate was centrifuged for 20 minutes at 14000 g and the supernatant was recovered for protein concentration quantification using the Pierce BCA Protein Assay Kit (Thermofisher Scientific). For subcellular fractionation, the kit NE-PER (Thermofisher Scientific) reagent was used on single germ cell suspension following manufacturer’s protocol. Proteins were resolved by molecular weight in on homemade 12.5% bis-acrylamide gels or 4–20% MINIPROTEAN TGX pre casted gels (BioRad) and transferred to methanol activated PVDF membranes using a Biorad Mini Trans-Blot Celby. After transfer, membranes were blocked using EveryBlot Blocking Buffer (BioRad) for 10 minutes at room temperature and incubated with rocking overnight at 4°C with diluted primary antibodies in EveryBlot Blocking Buffer (see S1 Resources for antibody information). After three consecutive washes in Tris-buffered saline with 0.1% Tween-20 (TBST) at room temperature for 5 minutes, membranes were incubated with secondary-HRP conjugated antibodies diluted in EveryBlot Blocking Buffer at room temperature for 2 hours. Finally, membranes were washed again three times in TBST and western blot signal was acquired with a Chemidoc Imaging System (BioRad).

### Prophase I chromosome spreads

Prophase I chromosome spreads were performed using the routine method employed by the Cohen Lab [50,80,81]. Testis were detunicated, and tubules were placed for 20 minutes on ice on hypotonic buffer (30 mM tris; 50 mM sucrose; 17 mM sodium citrate; 5 mM EDTA; 5 mM PMSF; 2.5 mM DTT; pH 8.2-8.4). Then, tubules were minced in a 500 mM solution of sucrose at room temperature, and the single cell suspension obtained was spread onto a slide previously coated with 1% paraformaldehyde with 0.15% Triton X (pH 9.2-9.3). The slides were slowly dried for 2–3 hours in a humid chamber and let air-dry for an extra hour. Then, they were washed with 0.4% Photo-flo 200 solution (Kodak Professional) for 5 minutes. Staining was done by washing the slides in 0.4% Photo-flo in PBS for 10 minutes, followed by permeabilization for 10 minutes in 0.1% Triton X in PBS, and blocking for 10 minutes in 10% antibody dilution buffer (3% bovine serum albumin, 10% normal goat serum, 0.0125% Triton X, in PBS). Finally, slides were incubated overnight with primary antibodies diluted in antibody dilution buffer in a humid chamber at room temperature. Secondary antibody incubation was performed the next day with previous Photo-flo washing, permeabilization and blocking steps described for primary antibodies. Slides were incubated with secondary antibodies at 37°C in a humid chamber for 2 hours, washed in PBS with 0.4% Photo-flo three times plus one in 0.4% Photo-flo in distilled water and mounted in antifade media with DAPI (2.3% DABCO, 20 mM Tris pH 8.0, 8 μg/ml DAPI in 90% glycerol). Slides were kept at 4°C until imaging on a Zeiss Axio Imager epifluorescence microscope equipped with Zeiss Zen Blue version 3.0 software (Carl Zeiss AG, Oberkochen, Germany). Images were processed using the Fiji software [82].

### Immunofluorescence and apoptosis detection on testicular histological sections

To obtain histological sections of testis from each genotype, testis were removed after euthanasia, one of them fixed in 10% formalin and the other one in Bouins solution at room temperature for 6 hours followed by three sequential 5-minute washes in 70% ethanol. Fixed testis were later embedded in paraffin and processed to obtain histological sections, 5 µm thick. Histological sections were deparaffinized with Safeclear xylene substitutes (Fisher Scientific) followed by decreasing ethanol concentrations. The slides were then gradually dehydrated by incubation in increasing concentrations of ethanol.

For immunofluorescence, antigen retrieval was done by immersing the slides on boiling citrate buffer (10mM sodium citrate, 0.05% Tween 20, pH 6) for 20 minutes, followed by 20 minutes incubation at room temperature. Then, slides were washed in PBS twice for 5 minutes. Sections were blocked in 0.05% Tween/PBS (PBST) with 1% bovine serum albumin and 3% goat serum for 1 hour at room temperature. After two washes with PBST for 5 minutes, sections were incubated with primary antibodies diluted in the blocking buffer at room temperature overnight. The next day, slides were washed three times in PBST and incubated with secondary antibodies diluted in blocking buffer for 2 hours at 37°C. Finally, sections were washed in PBST three times for 5 minutes and mounted in antifade media with DAPI (2.3% DABCO, 20 mM Tris pH 8.0, 8 μg/ml DAPI in 90% glycerol).

Apoptosis levels in testicular sections were evaluated with the commercial kit Apoptag kit (EMD Millipore) following the manufacturer’s instructions after deparaffinization. This kit detects DNA breaks associated with latest stages of apoptosis and is based on the dUTP nick-end labeling reaction of free 3’OH termini by terminal deoxynucleotidyl transferase (TdT), also known as TUNEL assay.

Testicular architecture was evaluated through Hematoxylin-eosin staining, followed by dehydration by sequential incubation in increasing concentrations of ethanol and mounting in toluene mounting media (Permount, Fisher Scientific). Hematoxylin-eosin stained and TUNEL sections were imaged using an Aperio CS2 Digital Pathology Slide Scanner microscope (Leica Biosystems) equipped with Aperio eSlide Manager Software. Immunofluorescent sections were imaged on a Zeiss Axio Imager epifluorescence microscope equipped with Zeiss Zen Blue version 3.0 software (Carl Zeiss AG, Oberkochen, Germany). Images were processed using the Fiji software [82].

### Testicular weight and sperm count

To study the reproductive phenotype in *Ago413* males, testicular weight and total body weight were recorded for each mouse after euthanasia. Both caudal epididymides were removed and placed in 1 mL of prewarmed and pH equilibrated DMEM containing 4% bovine serum albumin (Sigma-Aldrich). Sperm was allowed to swim out of the epididymis by incubation for 20 minutes at 37°C under 5% CO_2_ atmosphere. After this period, an aliquot of sperm was obtained and fixed in 10% formalin for quantification of total sperm using a hemocytometer.

### Enrichment on germ cells by Fluorescence activated cell sorting (FACS)

To prepare enriched germ cells fractions from each genotype for scRNA seq, we used Fluorescence activated cell sorting following the method developed by Rodriguez- Casuriaga et al [83]. First, a single cell suspension from testis was obtained following the method described by Ascenção et al [33]. Testis from 2-5 mice from the same genotype were collected, the *tunica albuginea* was removed, and tubules were placed in 10 ml of preheated (37°C) DMEM-F12 containing 2 mg of Collagenase 1A (Sigma) for 2 minutes with manual shaking. Digestion was stopped by two washes of the tubules with DMEM-F12. Next, the tubules were incubated in 10 ml of DMEM-F12 with 5 mg of trypsin (Sigma) and 7 mg/ml of DNAse I (Sigma) at 37°C with constant shaking (150 rpm) until the length of the tubules was about 1 mm (approximately 10 minutes). Digestion was stopped by addition of 3 ml of Fetal Bovine Serum (FBS, Sigma) and 30 ml of DMEM F-12. Digested tubules were strained on a 100 μm strainer and centrifuged at 15°C for 5 minutes at 600g. The single cell suspension of germ cells obtained was then resuspended in FBS and [31,81] incubated with 10 μM of Vybrant dye cycle (VDG) (Invitrogen) for 30 minutes at 37°C in the dark with constant rocking. VDG is DNA-specific vital stain that is fluorescent upon binding to double-stranded DNA and is excited at 488 nm with emission ~520 nm. Sorting of cells was performed at Cornell Flow Cytometer Facility (RRID:SCR_021740), using a Sony MA900 fluorescent activated cell sorter, tuned to emit at 488 nm and laser power set to 100 mW to collect for VDG positive cells. Flow cytometric profiles were obtained by representing Forward Scatter (Y axes) vs VDG fluorescence intensity (X axes), this allowed to identify different cell populations in the sample based on their DNA content and size. To enrich for spermatocytes for single cell RNA-seq, a sorting gate was set to collect all germ cell types excluding sperm [83]. Sorted cells were recovered in tubes containing DMEM-F12 buffer and 10% FBS. Enrichment in germ cells of the sorted cell populations was evaluated by performing immunofluorescence of meiotic spreads with the markers SYCP3, γH2AX and DAPI as previously described.

### Single Cell RNA sequencing library preparation

Single cell RNA sequencing libraries were prepared from enriched germ cell suspensions at the Transcriptional Regulation and Expression Facility at Cornell (RRID:SCR_022532) using the 10X Genomics Chromium Single Cell 3′ RNA-seq v3.1 kit. Flow sorted cells were processed on the 10X Genomics Chromium X System, targeting a total of 7000 cells per sample. Quality control was evaluated using an Agilent Fragment Analyzer and ran on a NovaSeqX platform with 150 base-pair reads.

### Single-cell transcriptome analysis

Fastq files were run through cellranger count (10x Genomics) [cellranger count –id = sampleID --r1-length = 28 --transcriptome = /path/to/refdata-gex-mm10–2020-A --fastqs = /path/to/directory --sample = file_prefix --localcores = 8 --localmem = 64] to generate count tables. Ambient RNA correction was then performed on samples using cellbender [cellbender remove-background --input/path/to/raw_feature_bc_matrix.h5 --output/path/to/output.hd5 --projected-ambient-count-threshold 0 --cpu-threads 32 --fpr 0.0 0.01 0.05 0.1 0.2 0.3 (--learning-rate 0.000025 for WT3; --learning-rate 0.00005 for KO1)]. Corrected samples with fpr 0.01 were imported into R to analyze with Seurat using Read_CellBender_h5_Multi_File (scCustomize). Cells were filtered based on number of genes detected, number of UMIs, and mitochondrial percentage, and cells with less than 500 genes or more than 9000 genes, less than 1500 UMIs, or more than 5% mitochondrial reads were removed from the analysis. Cell cycle scoring was performed (CellCycleScoring using Seurat genes and default parameters) to calculate the average expression level of S phase and G2/M genes compared to background, and these were used to calculate the difference between the S phase and G2M phase scores. Genes used for S phase scoring: *Mcm5, Pcna, Tyms, Fen1, Mcm2, Mcm4, Rrm1, Ung, Gins2, Mcm6, Cdca7, Dtl, Prim1, Uhrf1, Mlf1ip, Hells, Rfc2, Rpa2, Nasp, Rad51ap1, Gmnn, Wdr76, Slbp, Ccne2, Ubr7, Pold3, Msh2, Atad2, Rad51, Rrm2, Cdc45, Cdc6, Exo1, Tipin, Dscc1, Blm, Casp8ap2, Usp1, Clspn, Pola1, Chaf1b, Brip1 and E2f8*. Genes used for G2/M scoring: *Hmgb2, Cdk1, Nusap1, Ube2c, Birc5, Tpx2, Top2a, Ndc80, Cks2, Nuf2, Cks1b, Mki67, Tmpo, Cenpf, Tacc3, Fam64a, Smc4, Ccnb2, Ckap2l, Ckap2, Aurkb, Bub1, Kif11, Anp32e, Tubb4b, Gtse1, Kif20b, Hjurp, Cdca3, Hn1, Cdc20, Ttk, Cdc25c, Kif2c, Rangap1, Ncapd2, Dlgap5, Cdca2, Cdca8, Ect2, Kif23, Hmmr, Aurka, Psrc1, Anln, Lbr, Ckap5, Cenpe, Ctcf, Nek2, G2e3, Gas2l3, Cbx5 and Cenpa*. Cell counts were normalized (SCTransform, vars.to.regress = “CC.Difference”, return.only.var.genes = F) and used to run PCA analysis (default parameters). Batch correction was performed using RunHarmony and cells were then clustered (FindNeighbors; dims = 1:12, reduction = “harmony”, k.param = 30; FindClusters; resolution = 0.3, n.start = 100). Cluster cell types were manually identified using expression of known marker genes (*Cenpa*, *Stra8*, and *Dazl* for spermatogonia; *Dazl*, *Gm960*, and *Meiob* for leptotene and zygotene spermatocytes; *Psma8* and *Piwil* for pachytene; *Pou5f2* and *Ccna1* for diplotene and dividing spermatocytes; *Tex36*, *Sun5*, *Tssk1*, *Cstl1*, *Tnp1*, and *Prm1* for different stages of round spermatids; *Tnp1*, *Prm1*, lack of *Tssk1*, and lower *Cstl1* for elongating spermatids; and *Col1a2*, *Acta2*, *Vcam1*, *Insl3*, *Laptm5*, *Hbb-bt*, *Ptgds*, and *Wt1* for somatic cell types).

Doublets were then removed using DoubletFinder. To remove doublets, counts were normalized for each sample using SCTransform (default parameters) and then a PCA (default parameters) and UMAP (dims = 1:10) analysis were run. pK value for doublet removal was then chosen using paramSweep (PCs = 1:10, sct = TRUE), summarizeSweep, and find.pK (default parameters), with chosen pK of for WT1, for KO1, for WT2, for KO2, for WT3, and for KO3. Homotypic doublets were modeled using modelHomotypic with the previously identified cell types and doublet removal was then run (doubletFinder using parameters PCs = 1:10, pN = 0.25, pK = chosen pK, nExp = round(0.045*#cells), reuse.pANN = FALSE, sct = TRUE and then rerun reusing the pANN and nExp = round(round(0.045*#cells)*(1-homotypic proportion)) with other parameters the same). Once doublets were removed, the samples were renormalized using the same parameters, PCA was run (default parameters), and samples were integrated using Harmony (IntegrateLayers; method = HarmonyIntegration). Samples were clustered (FindNeighbors; dims = 1:25, reduction = “harmony”, k.param = 30; FindClusters; resolution = 0.3, n.start = 100). Cluster cell types were identified as previously described. One cluster showed very low expression of marker genes and expression of multiple marker genes which are not expressed in the same stage of cells and were removed. Normalization, PCA and integration were rerun on the remaining cells with the same parameters. Samples were reclustered (FindNeighbors; dims = 1:15, reduction = “harmony”, k.param = 30; FindClusters; resolution = 0.4, n.start = 100), for a total of 11 clusters, the UMAP projection was created (RunUMAP; dims = 1:15, reduction = “harmony”, n.neighbors = 30), and cluster cell types were identified and used in all following analysis. Cell type proportion changes were tested using sccomp [84] (sccomp_glm with parameters formula_composition = ~genotype and bimodal_mean_variability_association = TRUE). Scoring cells for meiotic regulation was done using the AddModuleScore function from Seurat and all genes in the GO term “GOBP_REGULATION_OF_MEIOTIC_CELL_CYCLE” (Genes: Aspm, Calr, Cdc20, Cdc25a, Cdc25b, Cdc25c, Chfr, Cntd1, Dmrt1, Dusp1, Fbxo43, Fbxo5, Fzr1, Gpr3, Hormad1, Insr, Lfng, Lif, Mapk15, Meioc, Meiosin, Mettl3, Mos, Msx1, Msx2, Nanos2, Npm2, Nppc, Npr2, Ooep, Ovol1, Pde3a, Piwil2, Plcb1, Prdm9, Psma8, Rad1, Rad51ap1, Rps6ka2, Sirt2, Spata22, Stra8, Topaz1, Trip13, Ttk, Ube2b, Wee2, Wnt4, Wnt5a, Ythdc2). The meiotic gene score was compared between groups using a two-sided Wilcoxon-rank sum test for significance. SCTransform normalized counts were used for mean expression levels by cell. Both values were visualized using ComplexHeatmap (parameters row_km = 5, row_km_repeats = 100). Chromosome X and chromosome 9 ratios were calculated for each cell by taking the mean SCTransform modified counts of all genes on either chromosome X or chromosome 9 and dividing by the mean expression of all autosomal genes. Ratios were visualized using ggplot and the gghalves package (geom_half_violin).

### WT cell type marker genes

Marker genes were identified for each cell type in wild-type cells (PrepSCTFindMarkers; FindAllMarkers, test.use = “MAST”, recorrect.umi = F). Marker gene sets for enrichment were then constructed by subsetting marker genes for adjusted p-value < 0.01 and average log2 fold-change >= 2 and then taking the top hundred genes ranked by adjusted p-value. Any genes that were duplicated between cell types were removed from the lists.

### Pseudobulk analysis

Cellbender corrected counts were aggregated by sample and cell type, as well as by cluster for cells identified as round spermatid 1 and 2. Counts were separated by cell type and filtered to remove genes with less than three samples having 10 or more counts. DESeq2 was used to normalize counts and to perform differential expression testing between knockout and wild-type samples with batch included in the design matrix for all samples and cluster also included for round spermatid 1 and 2 tests. The log_2_ fold-change results were used to run gene set enrichment analysis using clusterProfiler for GO terms (gseGO; ont = “BP”, OrgDb = org.Mm.e.g.,db, minGSSize = 50, pvalueCutoff = 0.05, verbose = FALSE, keyType = “ALIAS”) as well as for the marker genes identified for different cell types in wild-type cells (GSEA; minGSSize = 20, pvalueCutoff = 1, verbose = FALSE). All genes with adjusted p-value <0.05 and absolute value log_2_ fold-change > 0.5 were considered significantly differentially expressed for the DE gene barplots.

### Bulk RNA sequencing and small RNA sequencing

Bulk RNA sequencing and small RNA sequencing was performed in enriched fractions of pachytene spermatocytes and round spermatids obtained using a BSA gradient method developed by Da Ros and others [37]. Briefly, a single germ cell suspension was prepared from testis of each genotype following the published protocol and loaded into a 5-layer discontinuous bovine serum albumin density gradient for 2.5 hours to allow enrichment of the different cell populations by gravity sedimentation. The cell fractions were then manually collected, and cells were pelleted and washed in cold PBS by centrifugation. Purity of each fraction was evaluated by light microscopy and confirmed by immunofluorescence of meiotic spreads with the markers SYCP3 γH2AX and DAPI as previously described, and only fractions containing 70% to 80% purity for pachytene/diplotene spermatocytes and 80–90% purity for round spermatids were pooled. Finally, cells were resuspended in TRIzol LS (Thermo Fisher Scientific) and snap frozen for downstream RNA extraction with an extra chloroform extraction to remove residual phenol and addition of glyco-blue as a carrier to promote RNA precipitation. RNA integrity was assessed on an Agilent Fragment Analyzer.

Directional RNA-seq libraries were prepared from 100-200ng total RNA by the Transcriptional Regulation & Expression Facility at Cornell using the NEBNext Directional Ultra II RNA Library Prep Kit for Illumina (New England Biolabs), with initial polyA+ selection. Library quality was assessed on an Agilent Fragment Analyzer and libraries were sequenced on an Illumina Novaseq 6000 with 2x150bp reads.

Small RNA-seq libraries were prepared from 100ng total RNA by the Transcriptional Regulation & Expression Facility at Cornell using the NEBNext Small RNA Library Prep Kit for Illumina (New England Biolabs). Library quality was assessed on an Agilent Fragment Analyzer and libraries were sequenced on an Illumina NextSeq 500with 50 bp single end reads.

### Bulk RNA-seq analysis

Illumina pipeline software was used for base calling. Sequenced reads were trimmed for 3’ adaptor sequence and low-quality sequence and filtered to remove reads < 50nt with TrimGalore. Processed reads were mapped to the reference genome with STAR using --quantMode GeneCounts to generate raw counts per gene. Raw counts were then imported into R and subset by cell type for differential expression analysis. Any genes without at least 10 counts in at least 3 samples were pre-filtered and DESeq2 was used to normalize the data and compare expression between knockout and wild-type samples. The log_2_ fold-change values were used for gene set enrichment analysis using clusterProfiler with the wild-type scRNA-seq marker gene sets (GSEA; minGSSize = 20, pvalueCutoff = 1, verbose = FALSE). Genes with adjusted p-value <0.05 and absolute log_2_ fold-change > 0.5 were considered significantly differentially expressed for DE bar plots.

### smRNA-seq analysis

Illumina pipeline software was used for basecalling. Sequenced reads were trimmed for 3’ adaptor sequence and low-quality sequence and filtered to remove reads < 10nt with TrimGalore. Trimmed reads were processed using from miRDeep2 pipeline: collapsed to fasta format using fastq2fasta.pl, processed using mapper.pl, and mapped to miRBase [85] v22.1 mmu miRNA using quantifier.pl. Raw counts were imported into R and counts from individual miRNA were summed into families as defined by TargetScan Mouse v8 [86]. Counts tables were subset by cell type and miRNA families were removed that did not have at least 10 counts in at least 3 samples. DESeq2 was used to normalize the data and compare between knockout and wild-type samples. Log_2_ fold-changes and expression values were used in post-transcriptional regulatory analysis.

### Sperm motility and morphology

Sperm Motility was assessed using computer aided sperm analysis (CASA). An aliquot of sperm obtained by swim-out was collected as described above using TYH media [87] without sodium bicarbonate and or BSA and placed on a cytometry chamber mounted on a warmed stage (37°C). Sperm motility was recorded using 40X magnification and the percentage of motile sperm over total sperm per sample was calculated using software Hamilton Thorne HT CASA SCA. At least five different fields and 200 sperm cells were recorded. Sperm morphology was assessed in smears of fixed sperm with 1% paraformaldehyde and stained with hematoxylin and eosin [88]. A minimum of 250 sperm were visualized per mouse.

### Breeding assay, blastocyst collection and sexing of progeny

Homozygous wild-type, heterozygous and homozygous null *Ago413* males of 10 weeks old were housed to breed to CD-1 females. After natural mating, time to first litter, litter size and sex ratio were recorded. The breeding pair was kept together until they produced at least a total of three litters. For blastocyst collection, breeding pairs of *Ago413* males from the three genotypes and CD-1 females were housed together and females were checked for plugs every morning. Upon plugging, females were removed from the breeding and housed individually until 3.5 days post plug. At this point, females were euthanized, and the uterus was quickly dissected out and placed directly in a watch glass containing pre-warmed M2 media (MR-015-D, Sigma-Aldrich) in a heated dissection hood. Blastocysts were flushed from the uterus by inserting a 1ml syringe filled with M2 media into the lumen of the uterus just below the oviduct and pushing the fluid through the uterine horns and out the cervix. Blastocysts were collected from the watch glass, washed in a droplet of PBS and individually pipetted into PCR strip tubes and frozen at -80 °C. After thawing, DNA was extracted from blastocysts by using the Extract-N-Amp Tissue PCR Kit (XNAT2, Sigma-Aldrich). Female and male embryos were identified by PCR using primers designed flanking an 84 bp deletion of the X-linked *Rbm31x* gene relative to its Y-linked homolog *Rbm31y* as previously described [89].

### Digital Droplet PCR (ddPCR) of sperm DNA

Sperm DNA extraction was adapted from Cole and Jasin [90]. Spermatozoa was prepared using the swim up technique that allows to separate motile from non-motile spermatozoa based on its ability to swim upwards through an overlaid medium [91]. Swim up and non-swim up sperm were passed through a 70 µm filter washed four times with saline sodium citrate by centrifugation at 5000 rpm for 1 minute. Sperm was resuspended in 5% SDS, 50% β-mercaptoethanol, 0.002% of proteinase K, in sodium citrate buffer and incubated for 2 hours at 55 °C inverting occasionally. Following incubation, a volume of phenol: chloroform: isoamyl alcohol (25:24:1) was added to extract protein, and tubes were spun at 15000 xrcf for 5 minutes. The aqueous phase was then transferred into a new tube, and DNA was precipitated by adding ice cold ethanol. The sample was mixed well and spun at 15000 x rcf for 5 minutes, the supernatant was discarded, and the pellet was washed in 70% ethanol by repeating centrifugation and removing the supernatant with a pipette tip. The pellet was then resuspended in DNAse free water and washed in 1/10 volume of sodium acetate and 3 volumes of cold ethanol and centrifuged again. The pellet was then washed once again in 70% ethanol, centrifuged and the supernatant discarded. The pellet was then left to air dry before being suspended in 100uL 5mM Tris HCl and incubated for 1 hour at 55°C. Samples were stored at -20°C until digital droplet PCR (ddPCR). To target the Y and X chromosome, TaqMan Gene Expression Assay probes (Thermo Fisher Scientific) against the genes *Sry* with a FAM label and *Rbmx* with a VIC label were used respectively. ddPCR was carried out by the Genomics Facility at Cornell (RRID:SCR_021727). The ratio of VIC to FAM signal was calculated to give the ratio of X to Y bearing sperm present in each sample.

### DNA Fluorescence in-situ hybridization (FISH) on sperm

Sexing of sperm was performed by targeting the X and Y chromosome for Fluorescent in situ hybridization (FISH) using an adapted protocol from Whyte et al [92]. Sperm was collected by swim up procedure as previously described and fixed in pre-soaked slides in ethanol for 48 hs. Slides were then dehydrated in 80% methanol at -20 °C for 20 minutes and then again air-dried overnight at room temperature. A droplet of 10mM dithiothreitol (DTT) in 0.1M Tris-HCl was added to the slides and then microwaved for 15 seconds at 550 Watts. Next, slides were drained and 20 μL of 10mM lithium diiodosalicylate (LIS) and 1mM DTT diluted in 0.1 mM Tris-HCl was added to each well. Slides were then covered in parafilm, microwaved for 90 seconds at 550 Watts and then washed twice in 2X saline sodium citrate. Slides were then treated again with 80% methanol for 20 minutes at -20 °C and air dried at room temperature. X and Y chromosome probes (Empire Genomics MCEN-XY-10-GRRE) were added to the hybridization buffer (Empire Genomics) and denatured at 65°C for 10 minutes and then held at 37 °C for 30 minutes. Probes were then added to each well, coverslips were applied and sealed with rubber cement and then microwaved for 78 seconds at 1100 Watts. Slides were transferred to a 37 °C prewarmed humid chamber and left for 18 hours at 37 °C to complete hybridization. Following hybridization, slides were washed in a pre-warmed 45 °C citrate buffer for 5 minutes to loosen coverslips. Slides were then placed into a pre-warmed wash solution of 50% formamide with 50% sodium citrate and kept at 45 °C for 8 minutes, drained and mounted with DAPI antifade. Slides were imaged on a Zeiss Axio Imager epifluorescence microscope equipped with Zeiss Zen Blue version 3.0 software (Carl Zeiss AG, Oberkochen, Germany). At least 200 sperm cells were counted and classified as X- or Y- bearing sperm.

### *In-vitro* fertilization (IVF)

Three-week-old C57BL/6J wild-type females were super ovulated by intraperitoneal injection of 5IU pregnant mare serum gonadotropin (PMSG, Sigma), followed by 5 IU of human chorionic gonadotropin (HCG, Sigma Aldrich) 46 hours later. Ovulated females were humanely euthanized, and ovaries were dissected out and placed in a watch glass containing pre-warmed M2 media (Milipore-Sigma, MR-015). *Cumulus*-oocyte clusters (COCs) were removed from each ovary and placed in pre-warmed M2 media and dissociated using hyaluronidase. Sperm was obtained by swim out in pre-equilibrated HTF media (Milipore-Sigma, MR-070-D) on a 5% CO_2_, 5% O_2_ and 90% N_2_ incubator at 37 °C for 30 minutes. After this time, sperm were counted using a hemocytometer and an equal number of sperm from each male was added to a 250μl droplet of HTF to achieve a concentration of 2 x 10^6^ spermatozoa/ml. Dissociated COCs were then transferred along with minimal media to the droplet containing sperm and incubated at 37°C, 5% CO_2_, 5% O_2_ and 90% N_2_ for 4 hours. After this time, COCs were transferred to plates containing warmed pre- equilibrated KSOM media (Millipore-Sigma, MR-101) and analyzed for the presence of a second polar body.

### Post-transcriptional regulatory analysis

Spermatocyte and round spermatid miRNA sets for the targeting signature analysis were chosen by taking the top 25 expressed miRNA and the top 10 expressed miRNA that were differentially expressed from each bulk smRNA-seq differential expression analysis. For spermatogonia, leptotene/zygotene, pachytene, and diplotene/dividing cell types the spermatocyte miRNA set was used, and four round spermatids 1 and 2 and elongating spermatids the spermatid miRNA set was used. The targeting signature was tested using the log_2_ fold-change from the pseudobulk analysis by subtracting the mean log_2_ fold-change of targets (TargetscanMouse v8 total weighted context score for gene < -0.2) from the mean log_2_ fold-change of background genes (genes without any predicted target sites for miRNA family but at least one predicted target site for any other miRNA family). Targeting signature significance was tested using a Wilcoxon rank sum test. The same process was used for spermatocyte and round spermatid bulk RNA-seq testing. For spermatid development gene set and XY-linked gene targeting analysis log_2_ fold-change of miRNA targets as defined above were plotted with a background set of all genes in the spermatid development gene set without a target site for any of the miRNA families plotted in the CDF plot.

To run miRsift, first buildContextTable was run with modified code to include all miRNA families which are broadly conserved and all miRNA families which are expressed in the smRNA-seq data and Targetscan identifies as conserved or poorly conserved but confidently annotated. For each of the cell types, pseudobulk counts were imported and prepared for analysis using importSeq and then the linear regression was run using miRsiftAnalyze.

### Multiome cell preparation

Single cell suspensions from wild-type and *Ago413*^*-/-*^ testis were obtained following the method described by Ascenção et al [33] and previously described here for scRNA-seq cell preparation. Before proceeding with nuclei isolation, spermatozoa were removed from the germ cell suspension samples by negative selection Magnetic Activated Cell Sorting (MACS) using an antibody against the acrosomal protein ACRV1 coupled with anti-rabbit IgG MicroBeads (Miltenyi Biotec) and following the protocol provided by the manufacturer. Briefly, cells were labeled with anti ACRV1 antibody (1:50 concentration) by incubation of 10^8^ cells in MACS buffer (0.5% BSA, 2mM EDTA in PBS, pH7.2) for 15 minutes. Then, the labelled cells were incubated with anti-rabbit IgG MicroBeads for 20 minutes on ice and loaded into an LS Column (Miltenyi Biotec) previously rinsed with MACS buffer. After depletion of spermatozoa by retention in the column, cells were collected from the negative fraction by centrifugation at 1500rpm for 5 minutes at 4°C and proceeded with nuclei isolation using 10X Genomics Demonstrated Protocol CG000365 Nuclei Isolation for Single Cell Multiome ATAC + Gene Expression Sequencing.

### Multiome library preparation

Single nucleus multiome libraries were prepared from enriched nuclei germ cell suspensions at the Cornell University BRC Genomics Core Facility (RRID:SCR_021727) using the 10X Genomics Chromium Next GEM Single Cell Multiome ATAC + Gene Expression kit. Flow sorted cells were processed on the 10X Genomics Chromium X System, targeting a total of 10000 cells per sample. Quality control was evaluated using an Agilent Fragment Analyzer and ran on a NovaSeqX platform 25B flow cells with 150 base-pair reads.

### Multiome analysis

The data was aligned to the mm10 reference genome from 10x, refdata-cellranger-arc-mm10–2020-A-2.0.0, using cellranger arc and default parameters. Samples were then analyzed using ArchR [93]. Arrow files were created using the createArrowFiles function with mints of 4 and minFrags of 1000. Doublet scores were added to the cells using addDoubletScores, and cells passing the default threshold were removed before downstream analysis. RNA counts were added to the project using the import10xFeatureMatrix and addGeneExpressionMatrix functions. All cells in the ATAC dataset without a corresponding cell in the RNA data were removed, and gene scores were added to each cell using the addGeneScore function.

Dimensions were added using the addIterativeLSI function for both ATAC and RNA (depthCol = “Gex_nUMI” and varFeatures = 2500 for RNA). Dimensions were combined using the addCombinedDims functions, and UMAP was added using default parameters. Clusters were identified with the addClusters function using the combined LSI and a resolution of 0.2. Cluster identities were determined by getting the RNA counts per cluster using the getGroupSE function and using marker expression as described previously. Cells were then subset using subsetArchRProject to include only those cells which were later spermatogonia and spermatocytes. Adding of dimensions and clustering was repeated as above, except using a resolution of 0.5. Clusters identified as early spermatocytes were further subclustered as above with resolution of 0.3 to separate pre-leptotene, leptotene, and zygotene cells. Peaks were identified per cluster using the addGroupCoverages, addReproduciblePeakSet, and addPeakMatrix function. Exon and intron counts were obtained by using velocyto [94] with the run10x option and read into R for further use.

Mean gene accessibility scores for the autosomes or X and Y were calculated by taking the mean of all gene score values on the relevant chromosome. Mean gene expression scores were calculated as for gene accessibility scores using RNA counts. As the peak matrix is not scaled, mean promoter and distal peak accessibility was calculated by first normalizing the peak matrix to 10,000 counts per cell and then taking the mean accessibility of peaks annotated as promoter or distal by ArchR.

For differential accessibility testing, raw peak counts were first aggregated by cluster and sample, and those peaks with a count of at least 10 in at least two samples were used for further testing. Peaks were then tested for differential accessibility using DESeq2, with a p-value < 0.05 considered significantly differentially accessible. Differential expression testing was conducted in the same manner using aggregated raw RNA counts. The overrepresentation test was run using the enrichGO function from clusterProfiler using biological process terms and the closest gene to each less accessible peak with p-value<0.05 as the input.

Pseudo time values were identified using the addSlingShotTrajectories function from ArchR on the combined UMAP and starting from the spermatogonia cluster. Accessibility, expression, and cell type composition values were aggregated in sliding windows using the slide function from the slider package with before and after equal to 50. SE values were calculated by dividing the sliding window SD by the square root of 101. Accessibility and expression sliding windows are separated by genotype and cell type includes both genotypes.

### Immunoprecipitation and Mass Spectrometry (IP-MS)

Immunoprecipitation was done in germ cell enriched single cell suspensions obtained from mouse testis as previously described [80]. After removing the testicular *tunica albuginea*, the seminiferous tubules were minced in PBS and further dissociated by pipetting to release the germ cells. The tubules and cell suspension were then filtered through a 70 μm cell strainer and the cells were pelleted by centrifugation at 4°C for 5 minutes at 600g. The cell pellet was resuspended in protein lysis buffer (50 mM Tris, 0.2% NP-40, 150 mM NaCl, 5 mM EDTA) with EDTA-free Protease Inhibitor Cocktail (Sigma) and sonicated for 12 seconds at 23% amplitude in cycles of 0.4 seconds on and 0.2 seconds off. Finally, lysates were centrifuged at 4 °C for 20 minutes at 15,000 x g, and the supernatant collected in a new tube. Before immunoprecipitation, lysates were precleared by incubation with the corresponding magnetic beads with no antibody conjugated (see S1 Resources) for 1 hour at 4°C on a nutator. After this period, beads were pelleted using a magnet and the precleared lysate was transferred to a new tube. For AGO4 immunoprecipitation, a lysate made from C57/B6 germ cells was incubated with an anti AGO4 antibody overnight at 4 °C on a nutator and an anti IgG rabbit was used as a control. The next day, the lysate was incubated with 10 μg of Protein A Dynabeads (Invitrogen) for 2 hours at 4 °C on a nutator. For AGO3 immunoprecipitation, a lysate made from *Ago3*^*myc-flag/myc-flag*^ germ cells was incubated with 10 μg of anti-MYC antibody covalently bound to magnetic beads (Cell Signaling) overnight at 4 °C on a nutator. As a control, lysates from *Ago3*^*+/+*^ germ cells were incubated with the same concentration of MYC-beads. After antibody and bead incubations, the flow through was collected and beads were washed six times with cold lysis buffer by pipetting, followed by pelleting using a magnet. Following the final wash, beads were resuspended in elution buffer (100mM Tris, 1% SDS, 10mM DTT) and incubated at 65 °C for 15 minutes. After incubation, the beads were pelleted with a magnet and elution was collected and used for Western Blot and Mass Spectrometry Analysis and label-free quantitation (LFQ) by the Proteomics and Metabolomics Facility at Cornell (RRID:SCR_021743). Elutions of three replicates per each immunoprecipitation were analyzed by nano LC-MS/MS using an Orbitrap Fusion mass spectrometer (Thermo-Fisher Scientific, San Jose, CA) equipped with a nanospray Flex Ion Source using high energy collision dissociation (HCD) coupled with UltiMate3000 RSLCnano (Dionex, Sunnyvale, CA). Reconstituted samples were injected onto a PepMap C-18 RP nano trap column (3 μm, 100 μm x 20 μm, Dionex) with nanoViper fittings at a 20 μL/min flow rate for on-line desalting, and subsequently separated on a PepMap C-18 RP nano column (3 μm, 75 μm x 25 μm) and eluted in a 120 minutes gradient of 5–35% acetonitrile (CAN_ in 0.1% formic acid) at 200 nL/min. The Orbitrap Fusion was operated in a data-dependent acquisition mode using FT mass analyzer for one survey scan followed by three “Top Speed” data- dependent CID ion trap MS/MS scans with normalized collision energy of 30%. A dynamic exclusion window of 45 seconds was specified. Data were acquired using Xcalibur 3.0 operation software and Orbitrap Fusion 2.0 (Thermo-Fisher Scientific).

Raw spectra for each sample and replicate were processed and proteome databases searched using Proteome Discoverer 2.5 (PD 2.5, Thermo-Fisher Scientific) with the Sequest HT search engine. Identified peptides were filtered for a maximum of 1% false discovery rate (FDR) using the Percolator algorithm in PD2.5. Relative label-free quantification was done in PD 2.5 to calculate protein abundances. The number of peptide spectrum matches (PSMs) were summed to represent protein abundance. Abundance ratios were calculated based on pairwise ratios using the median calculated among replicates.

## Supporting information

S1 Appendix Supplementary Tables A-F. (XLSX)

S1 Resources Key Resource table. (DOCX)

S1 Fig *Ago413* and *Ago3* knockout mouse models. (A) Overview of *Ago413* knockout mouse model. (B) Overview of *Ago3* knockout mouse model. (C) PCR of testis cDNA from wild-type and *Ago3*^*-/-*^ from founder A1 and A2 using four different sets of primers targeting the regions in the Ago3 mRNA indicated with different colors. (D-F) Western Blots on testis lysates from wild-type, heterozygous and null *Ago413* mice using different anti-AGO antibodies: AGO4 (D) AGO1 (E) and AGO3 (F). 60 μg of protein was loaded per lane, GADPH was used as a loading control. All WB show several unspecific bands, being the expected AGO size (97 kDa), numbers at the right indicate weight marker (kDa). (G) Western Blots on testis germ cell protein lysates from wild-type and the two null *Ago3*^*-/-*^ lines (A1 and A2), 80 μg of protein was loaded per lane, GADPH was used as a loading control. All WB show several unspecific bands, being the expected AGO size (97 kDa), numbers at the right indicate weight marker (kDa).
(TIF)

S2 Fig (A) Western Blots using an anti- MYC antibody on germ cell protein lysates from whole cell, nuclear and cytoplasmic fractions of *Ago3 myc flag* homozygous mice, expected AGO size (97 kDa) indicated with an arrow. 60 μg of protein was loaded per lane, GADPH and Histone 4 were used as a loading control of cytoplasmic and nuclear fractions respectively. (B) Western Blot with anti-HA antibody on germ cell protein lysates from whole cell, nuclear and cytoplasmic protein lysates from *Ago2*^*ha/ha*^ and *Ago2*^*+/+*^ germ cells showing presence of AGO2-HA in both subcellular compartments, GADPH and SYCP3 were used as loading controls of cytoplasmic and nuclear fractions respectively. 60 μg of protein was loaded per lane, expected AGO size (97 kDa) indicated with an arrow. (C) Prophase I spreads of *Ago2*^*ha/ha*^ mice immunostained with SYCP3 and anti-HA antibody show diffuse AGO2 in the nucleus of spermatocytes. (D) Testicular sections of homozygous *Ago2*^*ha/ha*^ and *Ago2*^*+/+*^ mice immunostained with SYCP3 and HA antibodies and DAPI. Arrows point at presence of AGO2 in the spermatocyte cytoplasm. Enlarged images show lighter staining in the nucleus compared to the cytoplasm. (E) Testicular sections of homozygous *Ago3*^*myc-flag*^ and *Ago3*^*+/+*^ mice immunostained with SYCP3 and FLAG antibodies and DAPI. Arrows in the enlarged images point at presence of AGO3 in sex bodies, while asterisks show localization of AGO3 to round spermatids nucleus. Bars indicate 40 μm.
(TIF)

S3 Fig Macroscopic phenotype of *Ago3* and *Ago413* knockout males. (A) Testis weights relative to body weight for *Ago3*^*+/+*^ and A1 and A2 *Ago3*^*-/-*^ males, dots represent each mouse and bars represent mean ± SD, n = 6 for wild-type, n = 7 for A1 and n = 5 for A2. (B) Epididymal spermatozoa counts obtained by swim out for for *Ago3*^*+/+*^ and A1 and A2 *Ago3*^*-/-*^ males, dots represent counts for one male and bars represent the mean ± SD, n = 6 for wild-type, n = 7 for A1 and n = 5 for A2. (C) Hematoxylin-Eosin staining of *Ago413*^*+/+*^, A1 *Ago3*^*-/-*^and *Ago413*^*-/-*^ testicular sections showing testicular architecture, magnified in panels on the bottom with indication for spermatocytes (Sp) and round spermatids (RS). Bars indicate 40 μm. (D) Meiotic progression of A1 and A2 *Ago3*^*-/-*^ males calculated as the percentage of cells in each substage of prophase I obtained after meiotic scoring of chromosome spreads through SYCP3 immunostaining. Dots represent each replicate and bars represent the mean ± SD, n = 3 for wild-type and A1 line, n = 2 for A2 line. (E) Litter size and log2 of female/male pups ratio (F) produced after natural mating of wild-type and A1 and A2 heterozygous and knockout *Ago3* males with wild-type females. Dots represent each litter and bars represent mean ± SD, n = 14 for wild-type, n = 22 for A1 *Ago3*^*+/-*^, n = 6 for A1 *Ago3*^*-/-*^, n = 12 for A2 *Ago3*^*+/-*^ and n = 12 for A2 *Ago3*^*-/-*^. Litters coming from at least three breeding pairs. Dots represent each individual litter and bars represent the mean ± SD. Data were analyzed by ANOVA followed by Tukey test for multiple comparisons.
(TIF)

S4 Fig Single cell RNA-seq Supplementary Data. (A) Expression levels of markers of germ and somatic cell types separated by cell type and genotype. (B) Percentage of cells by genotype identified as each cell type by marker gene expression in scRNA-seq. Each dot represents an individual sample. Bars represent the mean ± SD, n = 3. Differential proportion p-values tested using sccomp package sum-constrained independent Beta-binomial distribution testing (* p < 0.05).
(TIF)

S5 Fig Gene Ontology gene set enrichment analysis. Dot plot showing normalized enrichment score (NES) of biological process gene ontology term gene set enrichment analysis by single cell cluster: (A) Spg, (B) Lep/Zy, (C) Pachy, (D) Diplo/M1/M2, (E) RS1 and (F) RS2. Log2FC values were produced by pseudo bulk differential expression analysis between genotypes and p-values are FDR adjusted.
(TIF)

S6 Fig Additional Meiotic Analysis for A1 and A2 *Ago3*^*-/-*^ males and *Ago413*^*-/-*^ males (A) Quantification of aberrant localization of γH2AX in A1 and A2 *Ago3*^*-/-*^ pachytene spermatocytes, (B) TOPBP1, (C) ATR and (D), SETX. Dots represent each data point and bars represent mean ± SD, n = 3 for wild-type, n = 3 for A1 and n = 2 for A2 *Ago3*^*-/-*^ males. (E) Analysis of synaptic defects in *Ago413*^*-/-*^ males by quantification of aberrant of SYCP1 and MLH1 counts (F) in pachytene spermatocytes. Bars represent mean ± SD, dots represent each replicate (n = 3). Data were analyzed by Kruskall-Wallis followed by Dunn’s test for multiple comparisons. G. Localization patterns for SYCP1 and MLH1 proteins along with SYCP3 in prophase I spreads of wild-type and *Ago413*^*-/-*^ spermatocytes. Dashed boxes indicate the sex chromosomes.
(TIF)

S7 Fig Posttranscriptional gene regulation in germ cells from *Ago413*^*+/+*^ and *Ago413*^*-/-*^ males. (A) MA plot for mature miRNA families identified in smRNA-seq for enriched spermatocytes. Significantly differentially expressed families have an absolute value log2 fold-change greater than or equal to 1 and an adjusted p-value less than 0.05. (B) As in (A) for round spermatids. (C) Scatter plot showing difference in mean log2 fold-change for miRNA targets (Targetscan cumulative weighted context score < -0.2) minus background genes on y-axis and log2 fold-change of miRNA family in bulk spermatocytes on x-axis. Color and shape legend in (F). RNA log2 fold-changes from pseudo bulk analysis of spermatogonia, leptotene/zygotene, pachytene, and diplotene and dividing cell types. Shape and size indicate if multiple test corrected p-value for Wilcoxon rank-sum test between miRNA target log2 fold-changes and background log2 fold-changes are less than 0.05. Spermatogonia through pachytene show small but significant upregulation of gene expression. Diplotene and dividing show no trend towards upregulation. (D) As in (C) for round spermatids 1 and 2 and elongating spermatids, with log2 fold-change of miRNA families coming from bulk round spermatid smRNA-seq. Round spermatid and elongating spermatid clusters don’t show pattern of upregulation of gene expression. (E) As in (C) for bulk RNA-seq analysis. miRNA log2 fold-change comes from corresponding bulk smRNA-seq dataset. Both spermatocytes and round spermatids show small but significant upregulation from some miRNAs.
(TIF)

S8 Fig Posttranscriptional regulation in *Ago413* males spermatogenesis. (A-B) Cumulative distribution plot of KO/WT log2 fold-change from diplotene pseudo bulk analysis (A) and bulk spermatocyte RNA-seq (B), with miRNA targets and background genes subset to only genes in GO term spermatid development. (C-D) As in (A-B) for XY-linked genes for diplotene pseudo bulk analysis (C) and bulk spermatocyte RNA-seq (D). (E) Scatter plot of miRsift results for each miRNA family tested in multiple linear regression with pseudo bulk spermatogonia. miRsift uses single linear regression to test miRNA families for their contribution to RNA-seq changes individually and then multiple linear regression with significant to consider the effect of other miRNAs (https://github.com/SRHilz/miRsift). X-axis is regression coefficient from multiple linear regression test and y-axis is log10 FDR value for each miRNA. Negative regression coefficient represents decreased repression of miRNA family targets and positive regression coefficient represents increased repression of miRNA family targets. Red line indicates FDR significance threshold of 0.05. (F) As in (E) for leptotene/zygotene cluster. (G) As in (E) for pachytene.
(TIF)

S9 Fig Multiomics data cell types. (A) UMAP of all meiotic/pre-meiotic cells in dataset labeled by clustering on combined RNA and ATAC signal. (B) UMAP hex plot of mean autosomal gene accessibility score of wild-type cells.
(TIF)

S10 Fig (A) Violin plot and bar plot displaying the CPM expression of XY genes at different stages of spermatogenesis. P-values calculated with two-sided Wilcoxon rank sum test, (*p < 0.05, **p < 0.01, ***p < 0.001). (B) Bar plot of percentage of genes tested for differential expression between knockout and wild-type that are significant with p-value <0.05 for spliced or unspliced reads. Negative values indicate decreased expression and positive values indicate increased expression.
(TIF)

S11 Fig (A-B) Violin plot and bar plot displaying the average accessibility of autosomal promoter (A) and distal (B) peaks at different stages of spermatogenesis separated by genotype. P-values calculated with two-sided Wilcoxon rank sum test, (*p < 0.05, **p < 0.01, ***p < 0.001).
(TIF)

S12 Fig Line and ribbon plot showing mean sliding windows using values for XY gene expression and promoter and distal peak accessibility, separated by genotype, along the pseudo time values. Bar plot of percentage of different cell types across pseudo time values for all genotypes. Pseudo time values were identified using the addSlingShotTrajectories function from ArchR on the combined UMAP and starting from the spermatogonia cluster.
(TIF)
