# Supplementary material for: Argonaute proteins orchestrate Meiotic Sex Chromosome Inactivation and timing of the spermatogenic transcriptional program
Source: PLoS Genet. 2026 Jun 29;22(6):e1012217. doi: 10.1371/journal.pgen.1012217 (PMC13336459; doi:10.1371/journal.pgen.1012217)
Supplement: S1 Resources — (DOCX) [file pgen.1012217.s002.docx]

**Key Resource table**

| REAGENT or RESOURCE | SOURCE | IDENTIFIER |  |  |
| --- | --- | --- | --- | --- |
| Antibodies | | |  |  |
| AGO4 | Cell Signaling | D10F10, #6913 |  |  |
| AGO1 | Cell Signaling | #9388 |  |  |
| AGO3 | Cell Signaling | D15D2 #5054 |  |  |
| pan AGO | Millipore | MABE56 |  |  |
| H4 | Abcam | ab7311 |  |  |
| GADPH | Proteintech | HRP-60004 |  |  |
| SYCP3 | Lenzi et al ^3^ | Homeade |  |  |
| SYCP3 | Abcam | ab97672 |  |  |
| phospho H2A.X (Ser139) | Millipore | 05-636 |  |  |
| TOPBP1 | Rendtlew et al ^90^ | Homeade |  |  |
| ATR | Cell signaling | 2790 |  |  |
| MLH1 | BD Biosciences | 550838 |  |  |
| SYCP1 | Abcam | ab15090 |  |  |
| RNA Pol2 | Millipore | 05-623 |  |  |
| SETX | Abcam | 220827 |  |  |
| IgG rabbit | Cell Signaling | #2729 |  |  |
| MYC | Cell Signaling | 71D10 #2278 |  |  |
| FLAG | Millipore | F1804 |  |  |
| Secondary rabbit antibody | Thermo Fisher Scientific | A-11034 |  |  |
| Secondary mouse antibody | Thermo Fisher Scientific | A-11017 |  |  |
| Secondary rabbit antibody | Thermo Fisher Scientific | A-11012 |  |  |
| Secondary mouse antibody | Thermo Fisher Scientific | A32742 |  |  |
| Chemical, peptides and recombinant proteins | | |  |  |
| MYC Magnetic beads | Cell signalling | 71D10 #91856 |  |  |
| Dynabeads, Protein A | Thermo Fisher Scientific | 10002D |  |  |
| DMEM: F12 | Gibco | 11765054 |  |  |
| Glutamax | Gibco | 35050061 |  |  |
| Heat Inactivated FBS | Gibco | 10437-028 |  |  |
| MEM non-essential aminoacids | Thermo Fisher Scientific | 11140050 |  |  |
| Trypsin | Millipore-Sigma | T4799 |  |  |
| Collagenase | Millipore- SIgma | C5138 |  |  |
| DNAse | Millipore-Sigma | DN25 |  |  |
| PMSF | Millipore-Sigma | 93482 |  |  |
| Roche Complete tablets | Millipore Sigma | 4693116001 |  |  |
| PFA | Millipore-Sigma | 158127 |  |  |
| Triton- X | Millipore-Sigma | 648463 |  |  |
| Photo-flo 200 solution | Kodak Professional | 1464510 |  |  |
| DTT | Millipore-SIgma | D0632 |  |  |
| PMSG | Millipore-Sigma | G 4527 |  |  |
| HCG | Millipore-Sigma | CG10 |  |  |
| KSOM | Millipore-Sigma | MR-101-D |  |  |
| X and Y chromosome probes | Empire Genomics | MCEN-XY-10-GRRE |  |  |
| EveryBlot Blocking Buffer | Biorad | 12010020 |  |  |
| Critical commercial assays | | |  | Mouse monoclonal, F1804 |
| Single Cell 3′ RNA-seq v3.1 kit | 10x Genomics |  |  |  |
| NEBNext Directional Ultra II RNA Library Prep Kit for Illumina | New England Biolabs |  |  |  |
| NEBNext Small RNA Library Prep Kit for Illumina | New England Biolabs |  |  |  |
| Deposited data | | |  |  |
| scRNA-seq of *Ago413-/-* and wild-type mouse germ cell suspensions | This paper | GEO: GSE284043 |  |  |
| RNA-seq and smRNA-seq of of Ago413-/- and wild-type mouse enriched spermatocytes and round spermatids | This paper | GEO: GSE284043 |  |  |
| MS Proteomics of AGO3 IP and AGO4 IP in mouse enriched germline extracts | This paper | PXD059391 |  |  |
| leChRO-seq data from enriched germ cell populations | Alexander et al ^52^ | GEO: GSE212120 |  |  |
| Mouse reference genome NCBI build 38, mm10 | Genome Reference Consortium | https://www.ncbi.nlm.nih.gov/grc/mouse |  |  |
| miRBase v22.1 | Kozomara et al ^82^ | RRID:SCR_003152 |  |  |
| vM23 10x Genomics prebuilt reference | 10x Genomics | https://cf.10xgenomics.com/supp/cell-exp/refdata-gex-mm10-2020-A.tar.gz |  |  |
| Experimental models: Commertial kits | | |  |  |
| TaqMan Gene Expression Assay probes | Thermo Fisher Scientific | FAM label and *Rbmx* with a VIC label |  |  |
| ApopTag Plus Peroxidase In Situ Apoptosis Kit | Millipore-Sigma | S7101 |  |  |
| Extract-N-Amp Tissue PCR Kit | Sigma-Aldrich | XNAT2, |  |  |
| Experimental models: Organisms/strains | | |  |  |
| B6/C57 | JAX | Strain #:000664 |  |  |
| Ago413 | JAX | Strain # JAX:014152 |  |  |
| Ago3 | This paper | NA |  |  |
| Ago3 tagged (*Ago3 ^myc-flag^*) | This paper | NA |  |  |
| Ago2 tagged (*Ago2 ^ha^*) | Sala et al ^28^ | NA |  |  |
| Oligonucleotides | | |  |  |
| Genotyping *Ago413* mouse line F | IDT | 5’ TGTCCTCCAGATCCGACCTT 3’ |  |  |
| Genotyping *Ago413* mouse line R | IDT | 5’TCCAGTTTTCCTAACCCGGC 3’ |  |  |
| Genotyping *Ago3* mouse line F | IDT | 5’GTGCTTGCTTGTAGGGGGAT 3’ |  |  |
| Genotyping *Ago3* mouse line R | IDT | 5’AACACCGGAAGGTCGTTTAGG 3’ |  |  |
| Ago3 cDNA Primer set 1 F | IDT | 5’CCTTTATACAGCCAATCCACTTC3’ |  |  |
| Ago3 cDNA Primer set 1 R | IDT | 5’GTCGGAGCACCACGTCAA3’ |  |  |
| Ago3 cDNA Primer set 2 F | IDT | 5’  GGCCACATAGTGAGACCTTATC  3’ |  |  |
| Ago3 cDNA Primer set 2 R | IDT | 5’  CTACACTTCCCTGCACAGATAC  3’ |  |  |
| Ago3 cDNA Primer set 3 F | IDT | 5’TTGGAAGAAGCGGCAACATC3’ |  |  |
| Ago3 cDNA Primer set 3 R | IDT | 5’GATAGTGTGAAGGACGGCTGG3’ |  |  |
| Ago3 cDNA Primer set 4 F | IDT | 5’GTGAAACGTGTGGGAGATACA3’ |  |  |
| Ago3 cDNA Primer set 4 R | IDT | 5’CCAGCATGGCTACAGAGATAAA3’ |  |  |
| Genotyping *Ago3 myc-flag* mouse line F | IDT | 5´TGTCTCTCCCGACTGTGCCTCT 3´ |  |  |
| Genotyping *Ago3 myc-flag* mouse line R | IDT | 5'CTCAGGGGACAACATCGGGGTA 3' |  |  |
| Sexing blastocysts F | IDT | 5'CACCTTAAGAACAAGCCAATACA 3' |  |  |
| Sexing blastocyst R | IDT | 5'GGCTTGTCCTGAAAACATTTGG 3' |  |  |
| Software and algorithms | | |  |  |
| R 4.3.2 | R Core Team | RRID:SCR_001905 |  |  |
| tidyverse 2.0.0 | Wickham et al ^91^ | RRID:SCR_019186 |  |  |
| ggpubr 0.6.0 | Kassambara A | RRID:SCR_021139 |  |  |
| ggplot2 3.4.4 | H Wickham | RRID:SCR_014601 |  |  |
| gghalves 0.1.4 | Tiedemann F | https://github.com/erocoar/gghalves |  |  |
| clusterProfiler 4.10.0 | Wu et al^92^ | RRID:SCR_016884 |  |  |
| ggnewscale 0.4.10 | Campitelli E | https://github.com/eliocamp/ggnewscale |  |  |
| org.Mm.eg.db 3.18.0 | Carlson M | https://bioconductor.org/packages/release/data/annotation/html/org.Mm.eg.db.html |  |  |
| cowplot 1.1.3 | Wilke C | RRID:SCR_018081 |  |  |
| lme4 1.1-35.1 | Bates et al^93^ | RRID:SCR_015654 |  |  |
| emmeans 1.10.0 | Lenth R | RRID:SCR_018734 |  |  |
| Polychrome 1.5.1 | Coombes et al ^94^ | https://github.com/cran/Polychrome |  |  |
| circlize 0.4.15 | Gu et al ^95^ | RRID:SCR_002141 |  |  |
| edgeR 1.14.1 | Chen et al ^96^ | RRID:SCR_012802 |  |  |
| Cellranger 7.0.0 | 10X Genomics | RRID:SCR023221 |  |  |
| Seurat 5.0.1 | Hao et al ^97^ | RRID:SCR_016341 |  |  |
| scCustomize 2.0.1 | Marsh SE | RRID:SCR_024675 |  |  |
| sccomp 1.6.0 | Mangiola et al ^81^ | https://github.com/MangiolaLaboratory/sccomp |  |  |
| ComplexHeatmap 2.18.0 | Gu et al ^98^ | RRID:SCR_017270 |  |  |
| harmony 1.2.0 | Korsunsky et al ^99^ | RRID:SCR_023543 |  |  |
| cellbender | Fleming et al ^100^ | RRID:SCR_025990 |  |  |
| DoubletFinder 2.0.4 | McGinnis et al ^101^ | RRID:SCR_018771 |  |  |
| DEseq2 1.42.0 | Love et al ^102^ | RRID:SCR_015687 |  |  |
| miRsift | Hilz S | https://github.com/SRHilz/miRsift |  |  |
| STAR 2.7.0f | Dobin et al ^103,104^ | RRID:SCR_004463 |  |  |
| Trim Galore 0.6.5dev | Kreuger F | RRID:SCR_011847 |  |  |
| cutadapt 4.9 | Martin M | RRID:SCR_011841 |  |  |
| miRDeep2 2.0.0.7 | Friedländer et al ^104^ | RRID:SCR_010829 |  |  |
| TargetScan Mouse v8.2 | McGeary et al ^83^ | RRID:SCR_010845 |  |  |
| featureCounts | Liao et al ^105^ | RRID:SCR_012919 |  |  |
| ShinyGO | Xijin Ge et al ^106^ | RRID:SCR_019213 |  |  |
| Zeiss Zen Blue version 3.0 | Carl Zeiss AG, Oberkochen, Germany). | NA |  |  |
| Fiji | Schindelin et al ^79^ | RRID:SCR_002285 |  |  |
| Graphad Prism 10.2.3 | GraphPad Software, Boston, Massachusetts USA, | NA |  |  |
| HT CASA SCA | Hamilton Thorne | NA |  |  |
