# Supplementary figures and images for: Argonaute proteins orchestrate Meiotic Sex Chromosome Inactivation and timing of the spermatogenic transcriptional program
Source: PLoS Genet. 2026 Jun 29;22(6):e1012217. doi: 10.1371/journal.pgen.1012217 (PMC13336459; doi:10.1371/journal.pgen.1012217)

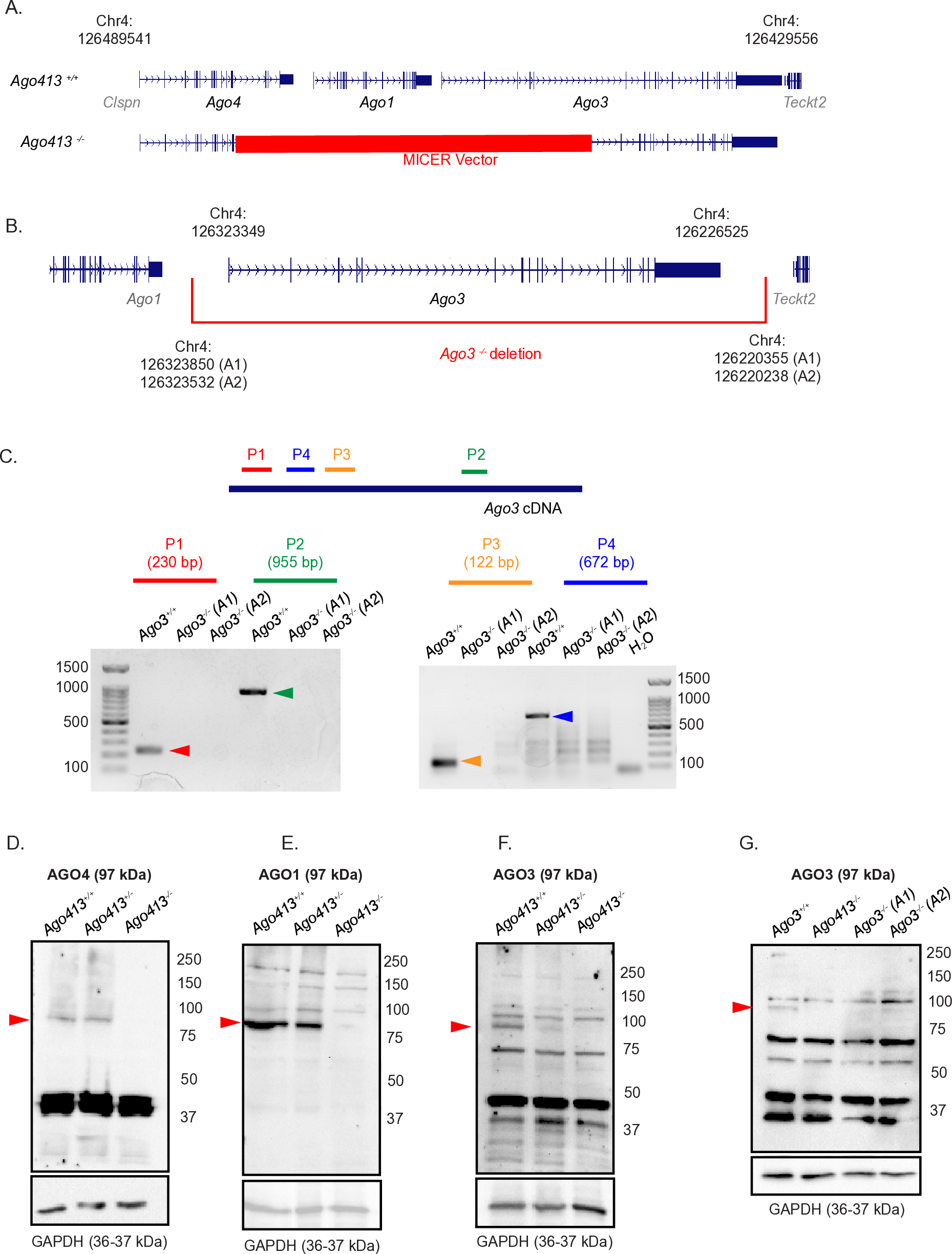

Supplement: S1 Fig — (A) Overview of Ago413 knockout mouse model. (B) Overview of Ago3 knockout mouse model. (C) PCR of testis cDNA from wild-type and Ago3-/- from founder A1 and A2 using four different sets of primers targeting the regions in the Ago3 mRNA indicated with different colors. (D-F) Western Blots on testis lysates from wild-type, heterozygous and null Ago413 mice using different anti-AGO antibodies: AGO4 (D) AGO1 (E) and AGO3 (F). 60 μg of protein was loaded per lane, GADPH was used as a loading control. All WB show several unspecific bands, being the expected AGO size (97 kDa), numbers at the right indicate weight marker (kDa). (G) Western Blots on testis germ cell protein lysates from wild-type and the two null Ago3-/- lines (A1 and A2), 80 μg of protein was loaded per lane, GADPH was used as a loading control. All WB show several unspecific bands, being the expected AGO size (97 kDa), numbers at the right indicate weight marker (kDa). (TIF) [file pgen.1012217.s003.tif]

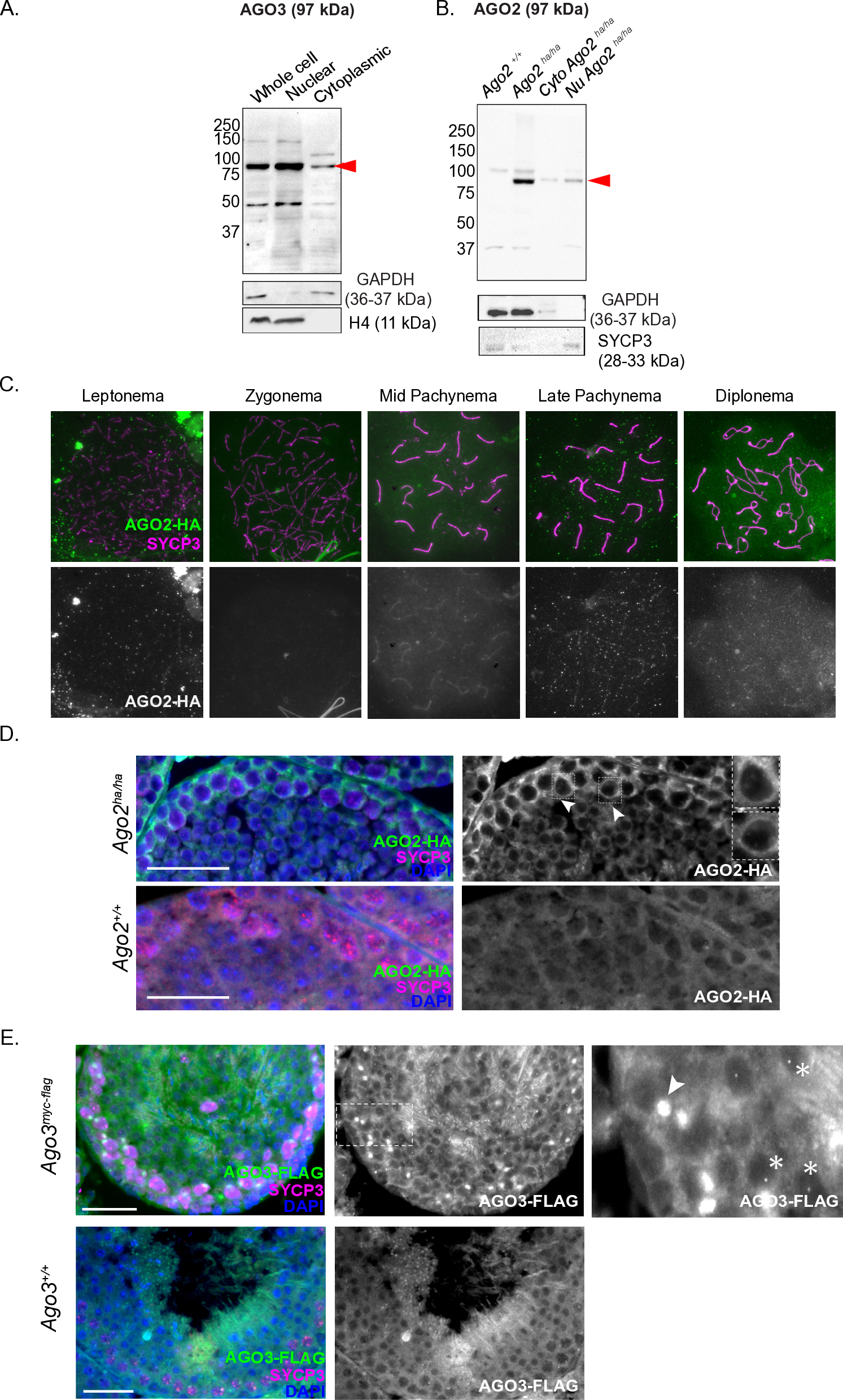

Supplement: S2 Fig — 60 μg of protein was loaded per lane, GADPH and Histone 4 were used as a loading control of cytoplasmic and nuclear fractions respectively. (B) Western Blot with anti-HA antibody on germ cell protein lysates from whole cell, nuclear and cytoplasmic protein lysates from Ago2ha/ha and Ago2+/+ germ cells showing presence of AGO2-HA in both subcellular compartments, GADPH and SYCP3 were used as loading controls of cytoplasmic and nuclear fractions respectively. 60 μg of protein was loaded per lane, expected AGO size (97 kDa) indicated with an arrow. (C) Prophase I spreads of Ago2ha/ha mice immunostained with SYCP3 and anti-HA antibody show diffuse AGO2 in the nucleus of spermatocytes. (D) Testicular sections of homozygous Ago2ha/ha and Ago2+/+ mice immunostained with SYCP3 and HA antibodies and DAPI. Arrows point at presence of AGO2 in the spermatocyte cytoplasm. Enlarged images show lighter staining in the nucleus compared to the cytoplasm. (E) Testicular sections of homozygous Ago3myc-flag and Ago3+/+ mice immunostained with SYCP3 and FLAG antibodies and DAPI. Arrows in the enlarged images point at presence of AGO3 in sex bodies, while asterisks show localization of AGO3 to round spermatids nucleus. Bars indicate 40 μm. (TIF) [file pgen.1012217.s004.tif]

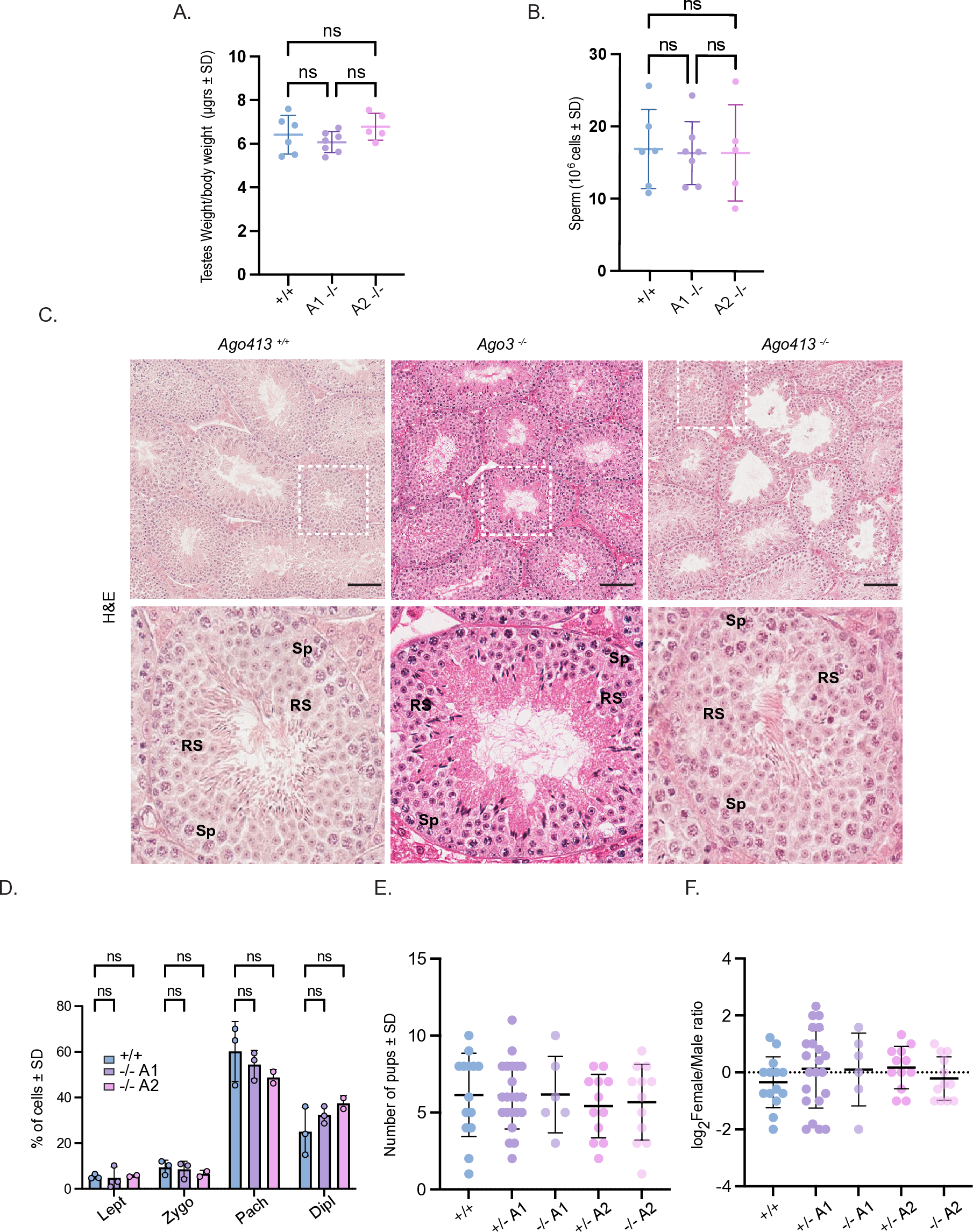

Supplement: S3 Fig — (A) Testis weights relative to body weight for Ago3+/+ and A1 and A2 Ago3-/- males, dots represent each mouse and bars represent mean ± SD, n = 6 for wild-type, n = 7 for A1 and n = 5 for A2. (B) Epididymal spermatozoa counts obtained by swim out for for Ago3+/+ and A1 and A2 Ago3-/- males, dots represent counts for one male and bars represent the mean ± SD, n = 6 for wild-type, n = 7 for A1 and n = 5 for A2. (C) Hematoxylin-Eosin staining of Ago413+/+, A1 Ago3-/-and Ago413-/- testicular sections showing testicular architecture, magnified in panels on the bottom with indication for spermatocytes (Sp) and round spermatids (RS). Bars indicate 40 μm. (D) Meiotic progression of A1 and A2 Ago3-/- males calculated as the percentage of cells in each substage of prophase I obtained after meiotic scoring of chromosome spreads through SYCP3 immunostaining. Dots represent each replicate and bars represent the mean ± SD, n = 3 for wild-type and A1 line, n = 2 for A2 line. (E) Litter size and log2 of female/male pups ratio (F) produced after natural mating of wild-type and A1 and A2 heterozygous and knockout Ago3 males with wild-type females. Dots represent each litter and bars represent mean ± SD, n = 14 for wild-type, n = 22 for A1 Ago3+/-, n = 6 for A1 Ago3-/-, n = 12 for A2 Ago3+/- and n = 12 for A2 Ago3-/-. Litters coming from at least three breeding pairs. Dots represent each individual litter and bars represent the mean ± SD. Data were analyzed by ANOVA followed by Tukey test for multiple comparisons. (TIF) [file pgen.1012217.s005.tif]

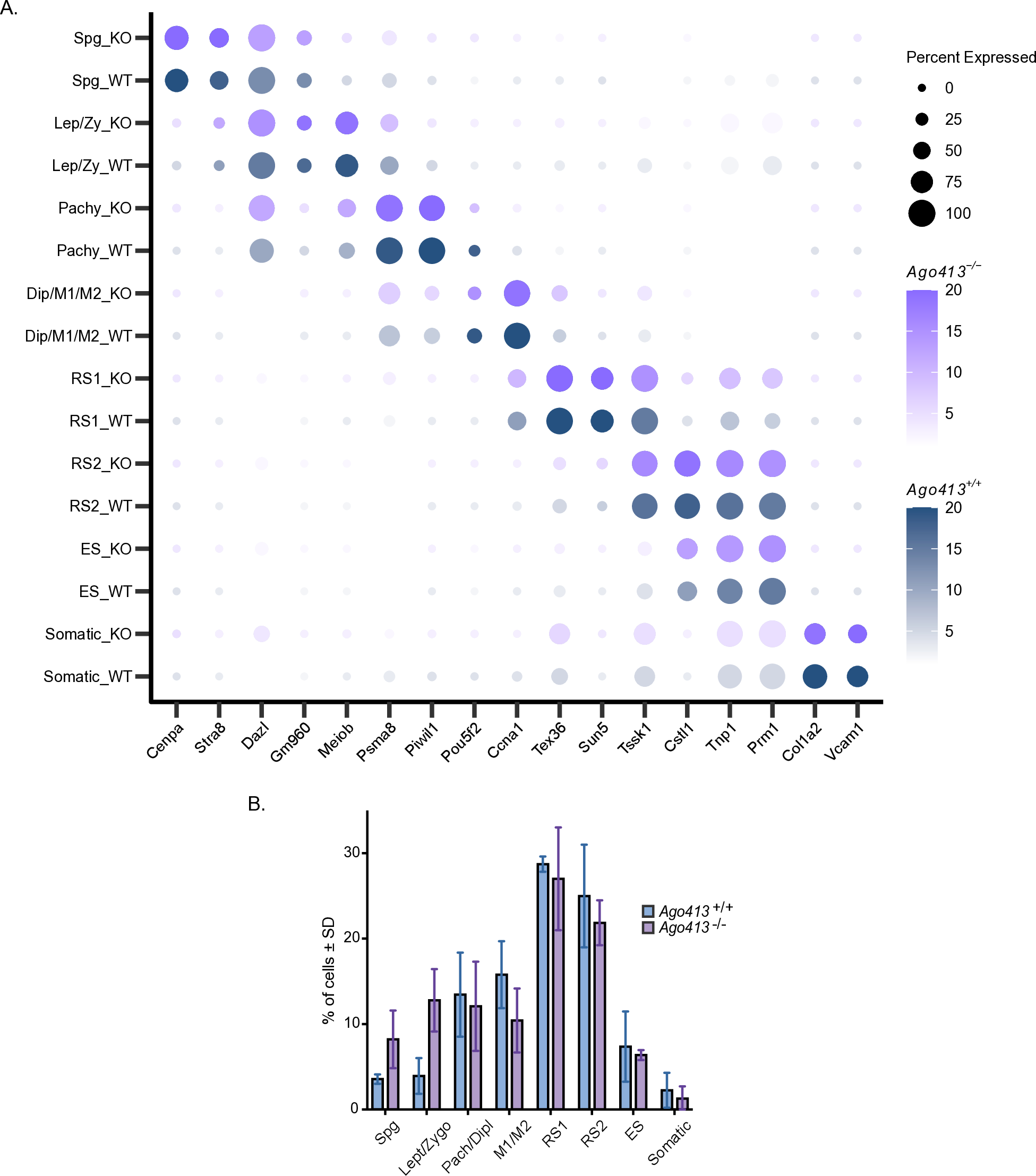

Supplement: S4 Fig — (A) Expression levels of markers of germ and somatic cell types separated by cell type and genotype. (B) Percentage of cells by genotype identified as each cell type by marker gene expression in scRNA-seq. Each dot represents an individual sample. Bars represent the mean ± SD, n = 3. Differential proportion p-values tested using sccomp package sum-constrained independent Beta-binomial distribution testing (* p < 0.05). (TIF) [file pgen.1012217.s006.tif]

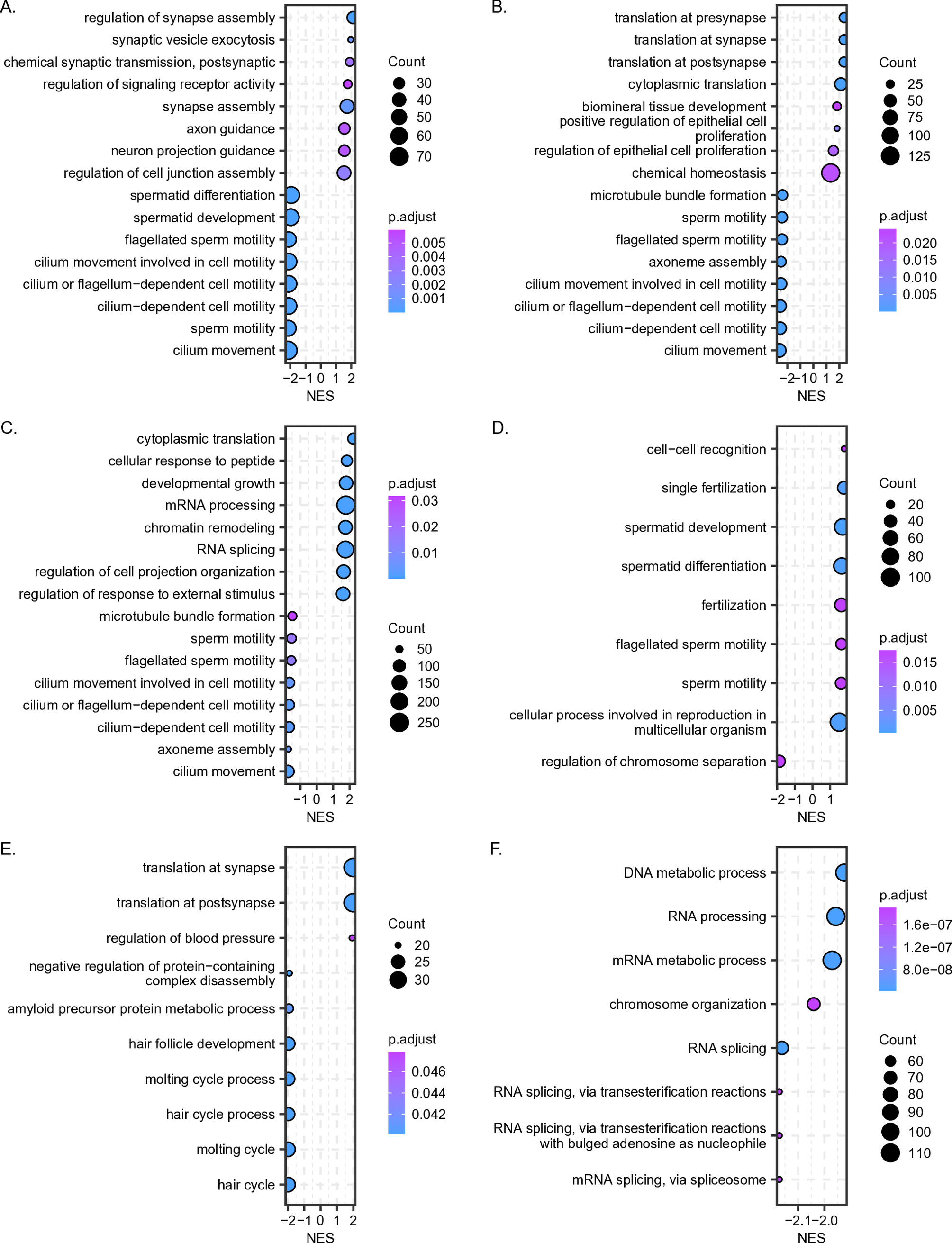

Supplement: S5 Fig — Dot plot showing normalized enrichment score (NES) of biological process gene ontology term gene set enrichment analysis by single cell cluster: (A) Spg, (B) Lep/Zy, (C) Pachy, (D) Diplo/M1/M2, (E) RS1 and (F) RS2. Log2FC values were produced by pseudo bulk differential expression analysis between genotypes and p-values are FDR adjusted. (TIF) [file pgen.1012217.s007.tif]

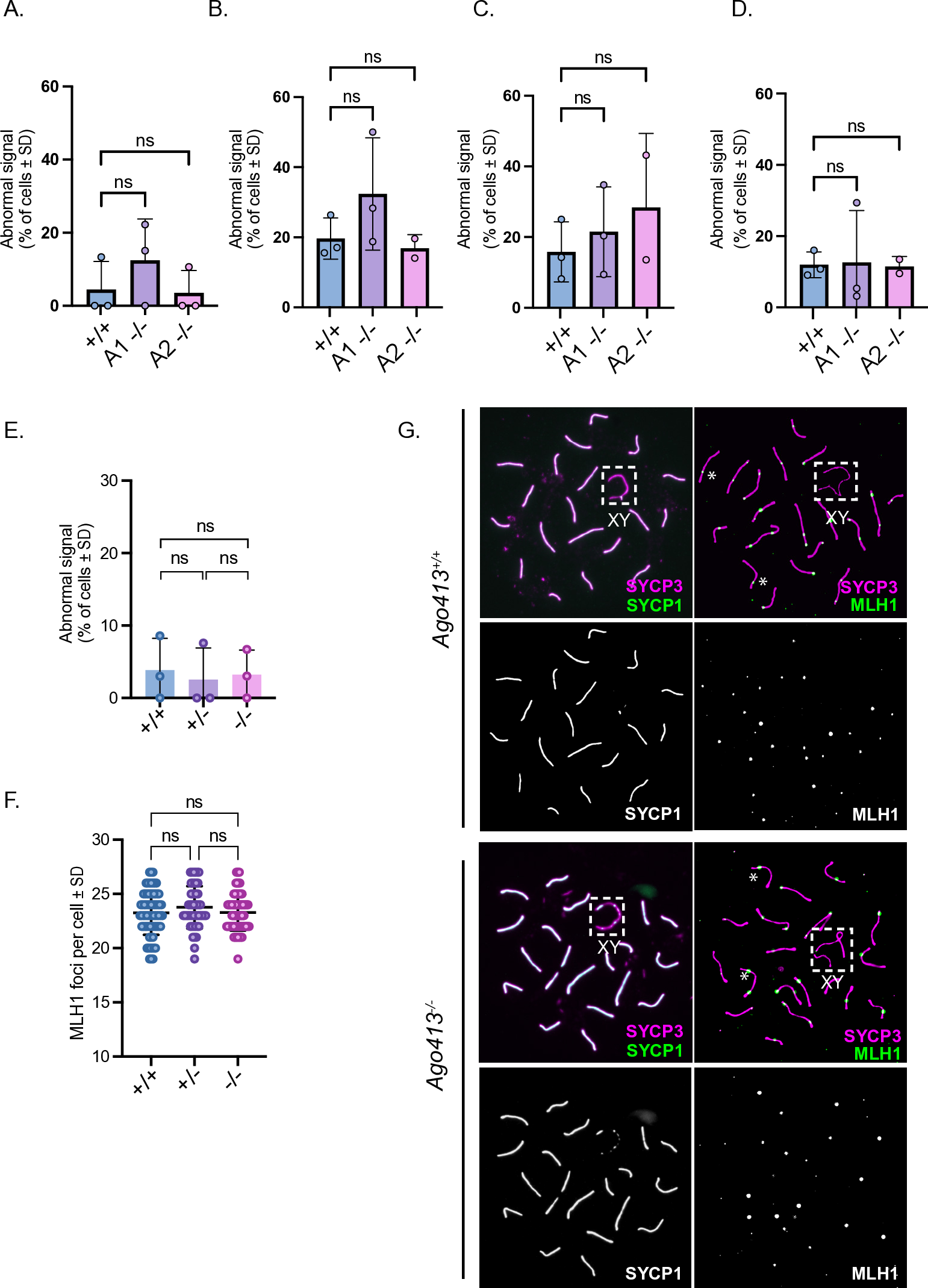

Supplement: S6 Fig — Dots represent each data point and bars represent mean ± SD, n = 3 for wild-type, n = 3 for A1 and n = 2 for A2 Ago3-/- males. (E) Analysis of synaptic defects in Ago413-/- males by quantification of aberrant of SYCP1 and MLH1 counts (F) in pachytene spermatocytes. Bars represent mean ± SD, dots represent each replicate (n = 3). Data were analyzed by Kruskall-Wallis followed by Dunn’s test for multiple comparisons. G. Localization patterns for SYCP1 and MLH1 proteins along with SYCP3 in prophase I spreads of wild-type and Ago413-/- spermatocytes. Dashed boxes indicate the sex chromosomes. (TIF) [file pgen.1012217.s008.tif]

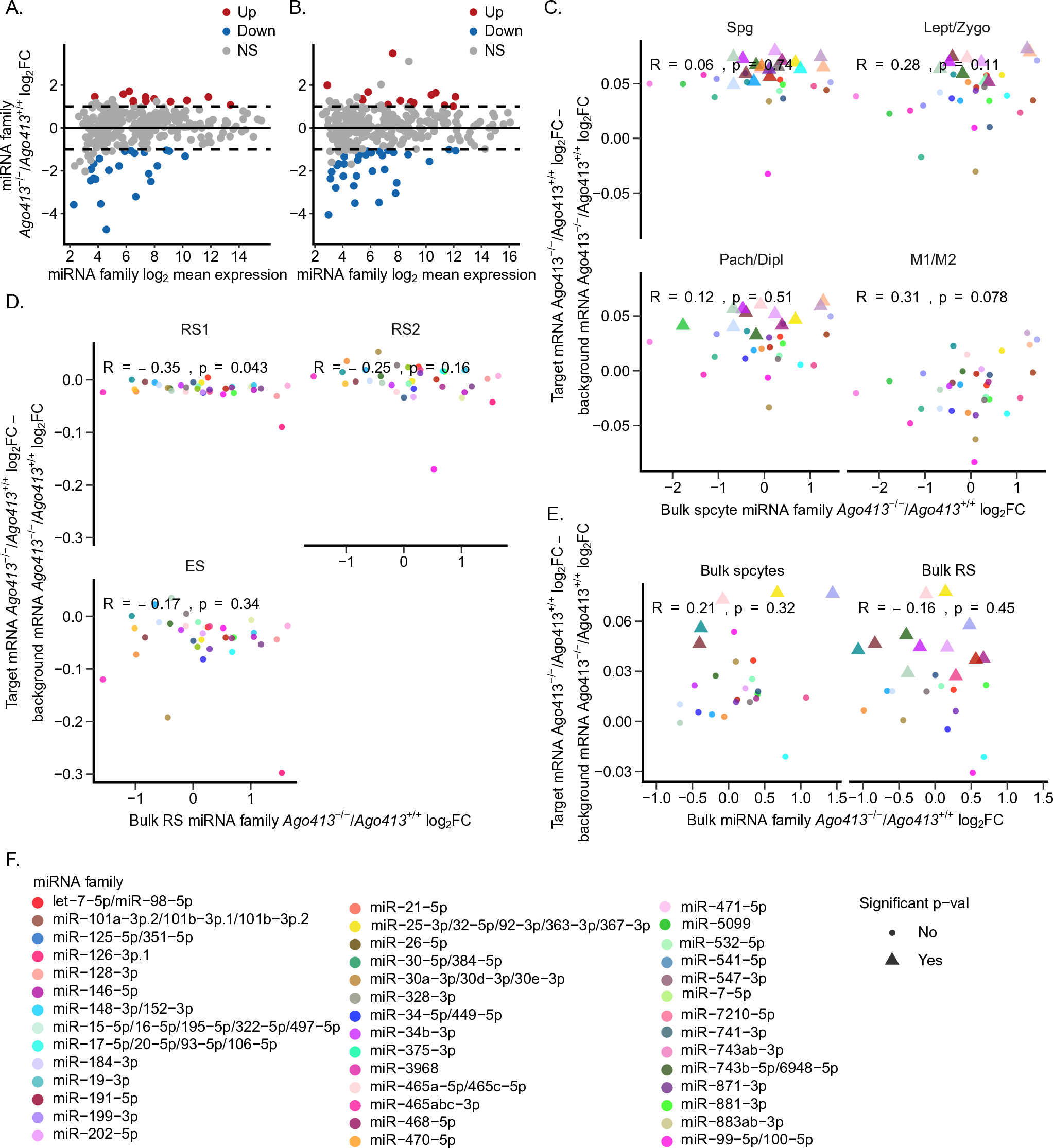

Supplement: S7 Fig — (A) MA plot for mature miRNA families identified in smRNA-seq for enriched spermatocytes. Significantly differentially expressed families have an absolute value log2 fold-change greater than or equal to 1 and an adjusted p-value less than 0.05. (B) As in (A) for round spermatids. (C) Scatter plot showing difference in mean log2 fold-change for miRNA targets (Targetscan cumulative weighted context score < -0.2) minus background genes on y-axis and log2 fold-change of miRNA family in bulk spermatocytes on x-axis. Color and shape legend in (F). RNA log2 fold-changes from pseudo bulk analysis of spermatogonia, leptotene/zygotene, pachytene, and diplotene and dividing cell types. Shape and size indicate if multiple test corrected p-value for Wilcoxon rank-sum test between miRNA target log2 fold-changes and background log2 fold-changes are less than 0.05. Spermatogonia through pachytene show small but significant upregulation of gene expression. Diplotene and dividing show no trend towards upregulation. (D) As in (C) for round spermatids 1 and 2 and elongating spermatids, with log2 fold-change of miRNA families coming from bulk round spermatid smRNA-seq. Round spermatid and elongating spermatid clusters don’t show pattern of upregulation of gene expression. (E) As in (C) for bulk RNA-seq analysis. miRNA log2 fold-change comes from corresponding bulk smRNA-seq dataset. Both spermatocytes and round spermatids show small but significant upregulation from some miRNAs. (TIF) [file pgen.1012217.s009.tif]

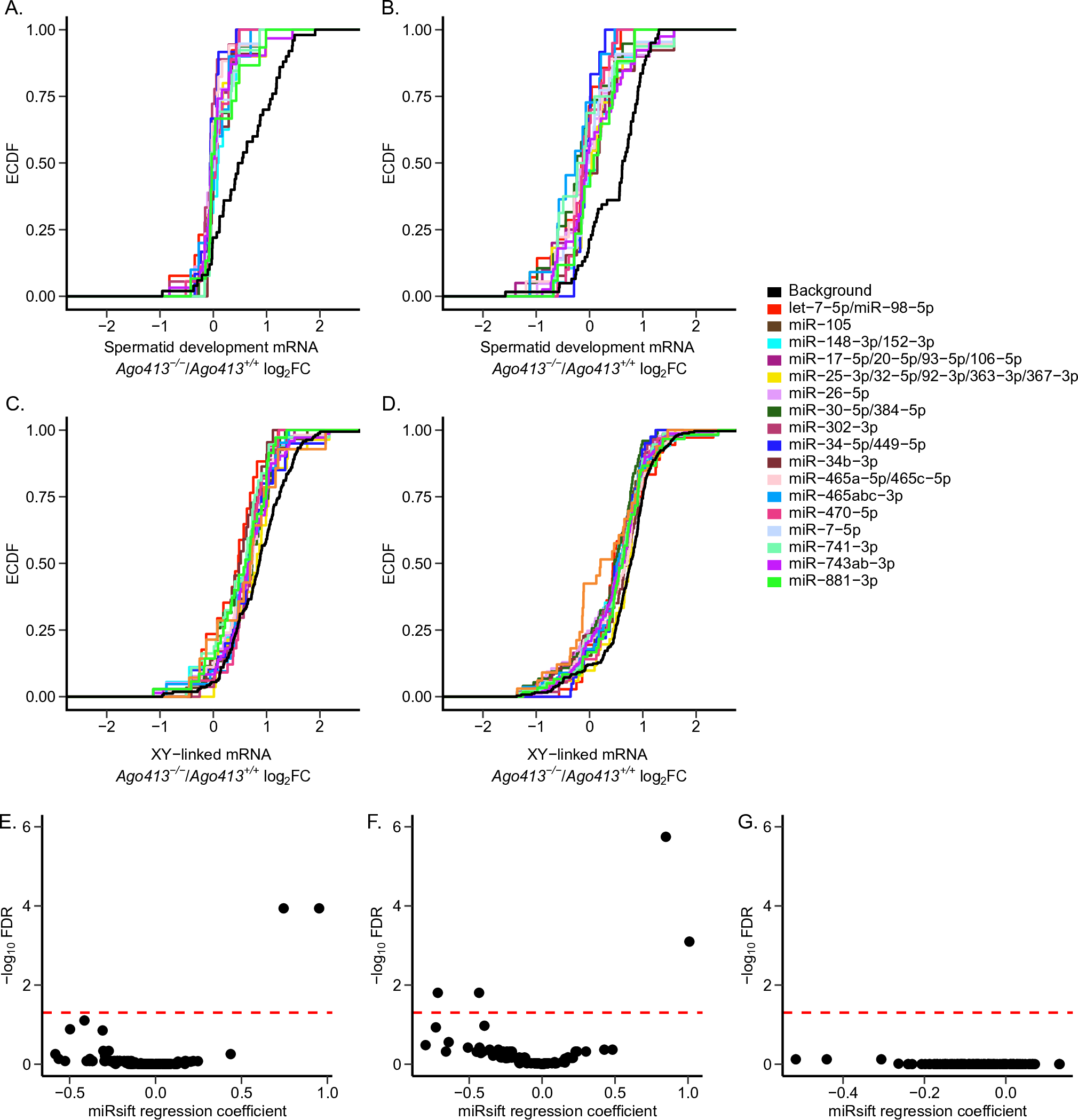

Supplement: S8 Fig — (A-B) Cumulative distribution plot of KO/WT log2 fold-change from diplotene pseudo bulk analysis (A) and bulk spermatocyte RNA-seq (B), with miRNA targets and background genes subset to only genes in GO term spermatid development. (C-D) As in (A-B) for XY-linked genes for diplotene pseudo bulk analysis (C) and bulk spermatocyte RNA-seq (D). (E) Scatter plot of miRsift results for each miRNA family tested in multiple linear regression with pseudo bulk spermatogonia. miRsift uses single linear regression to test miRNA families for their contribution to RNA-seq changes individually and then multiple linear regression with significant to consider the effect of other miRNAs (https://github.com/SRHilz/miRsift). X-axis is regression coefficient from multiple linear regression test and y-axis is log10 FDR value for each miRNA. Negative regression coefficient represents decreased repression of miRNA family targets and positive regression coefficient represents increased repression of miRNA family targets. Red line indicates FDR significance threshold of 0.05. (F) As in (E) for leptotene/zygotene cluster. (G) As in (E) for pachytene. (TIF) [file pgen.1012217.s010.tif]

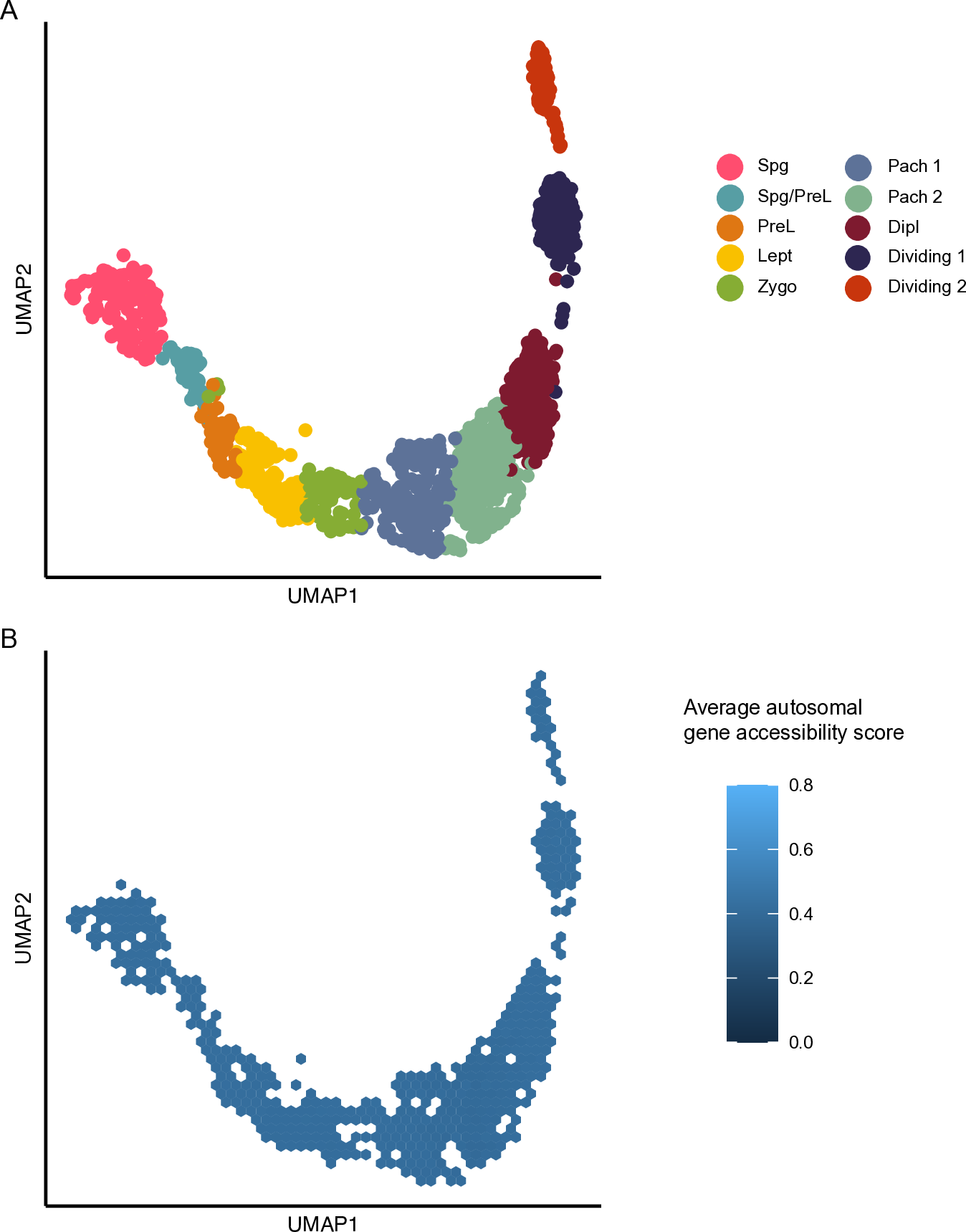

Supplement: S9 Fig — (A) UMAP of all meiotic/pre-meiotic cells in dataset labeled by clustering on combined RNA and ATAC signal. (B) UMAP hex plot of mean autosomal gene accessibility score of wild-type cells. (TIF) [file pgen.1012217.s011.tif]

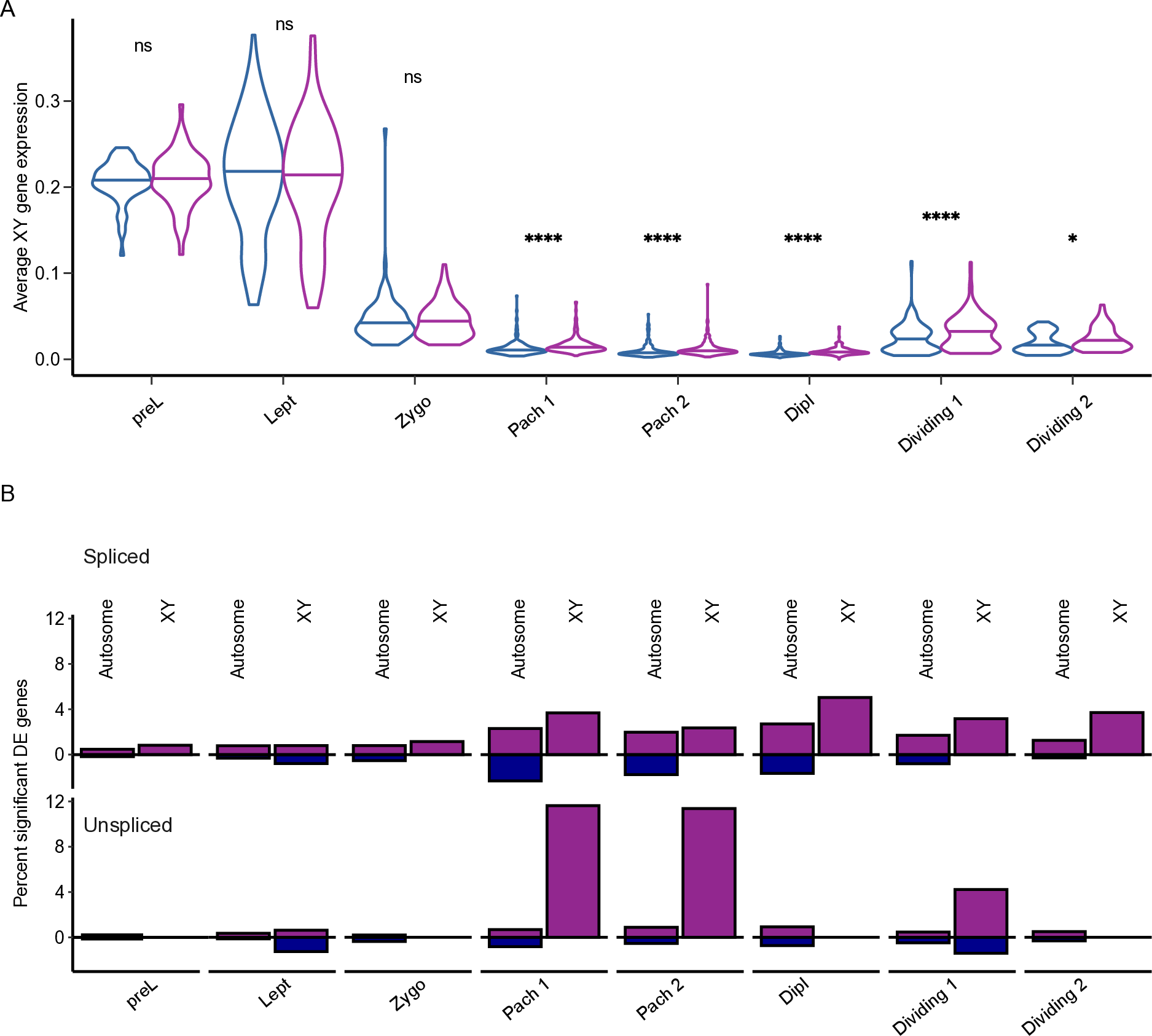

Supplement: S10 Fig — P-values calculated with two-sided Wilcoxon rank sum test, (*p < 0.05, **p < 0.01, ***p < 0.001). (B) Bar plot of percentage of genes tested for differential expression between knockout and wild-type that are significant with p-value <0.05 for spliced or unspliced reads. Negative values indicate decreased expression and positive values indicate increased expression. (TIF) [file pgen.1012217.s012.tif]

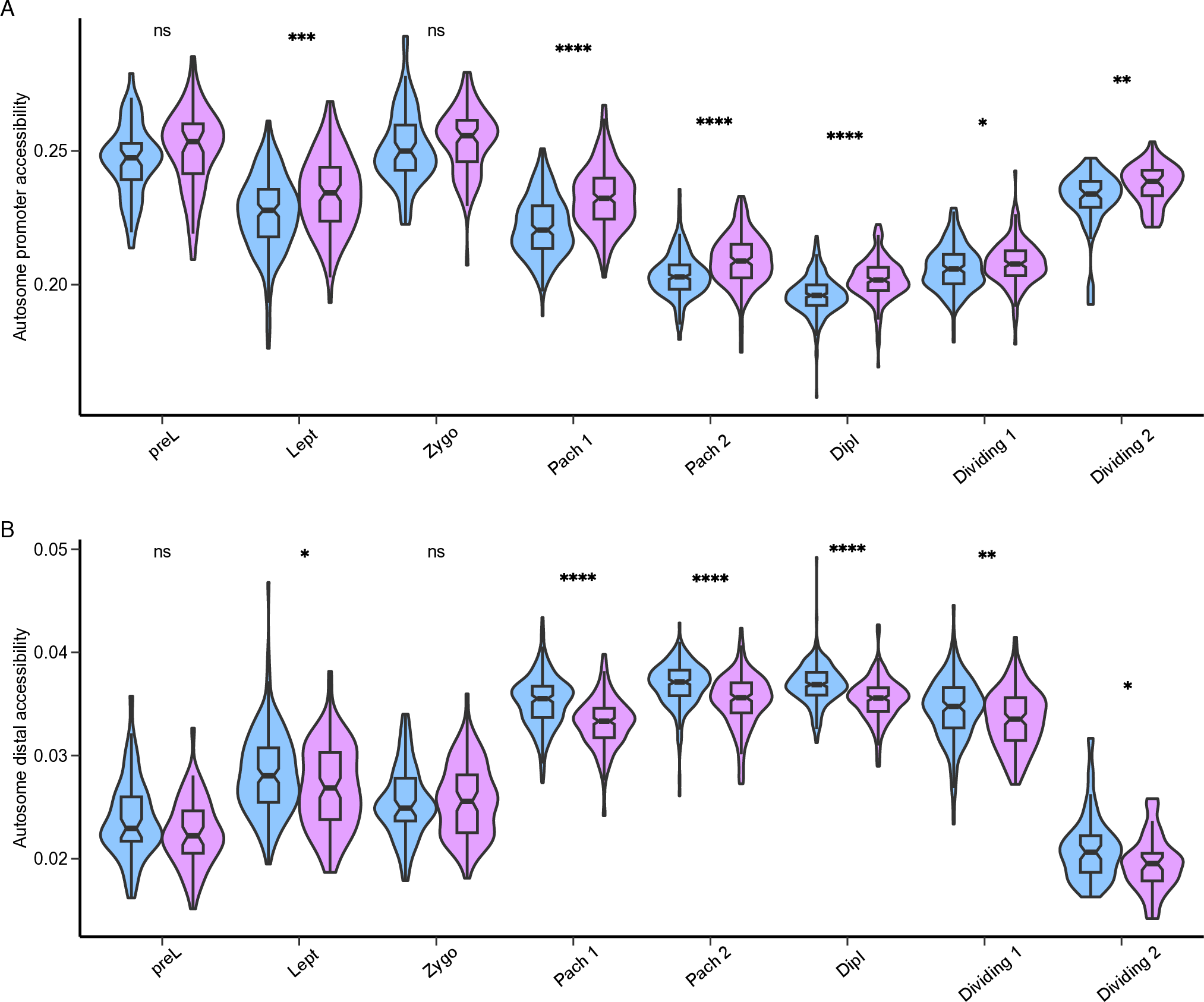

Supplement: S11 Fig — P-values calculated with two-sided Wilcoxon rank sum test, (*p < 0.05, **p < 0.01, ***p < 0.001). (TIF) [file pgen.1012217.s013.tif]

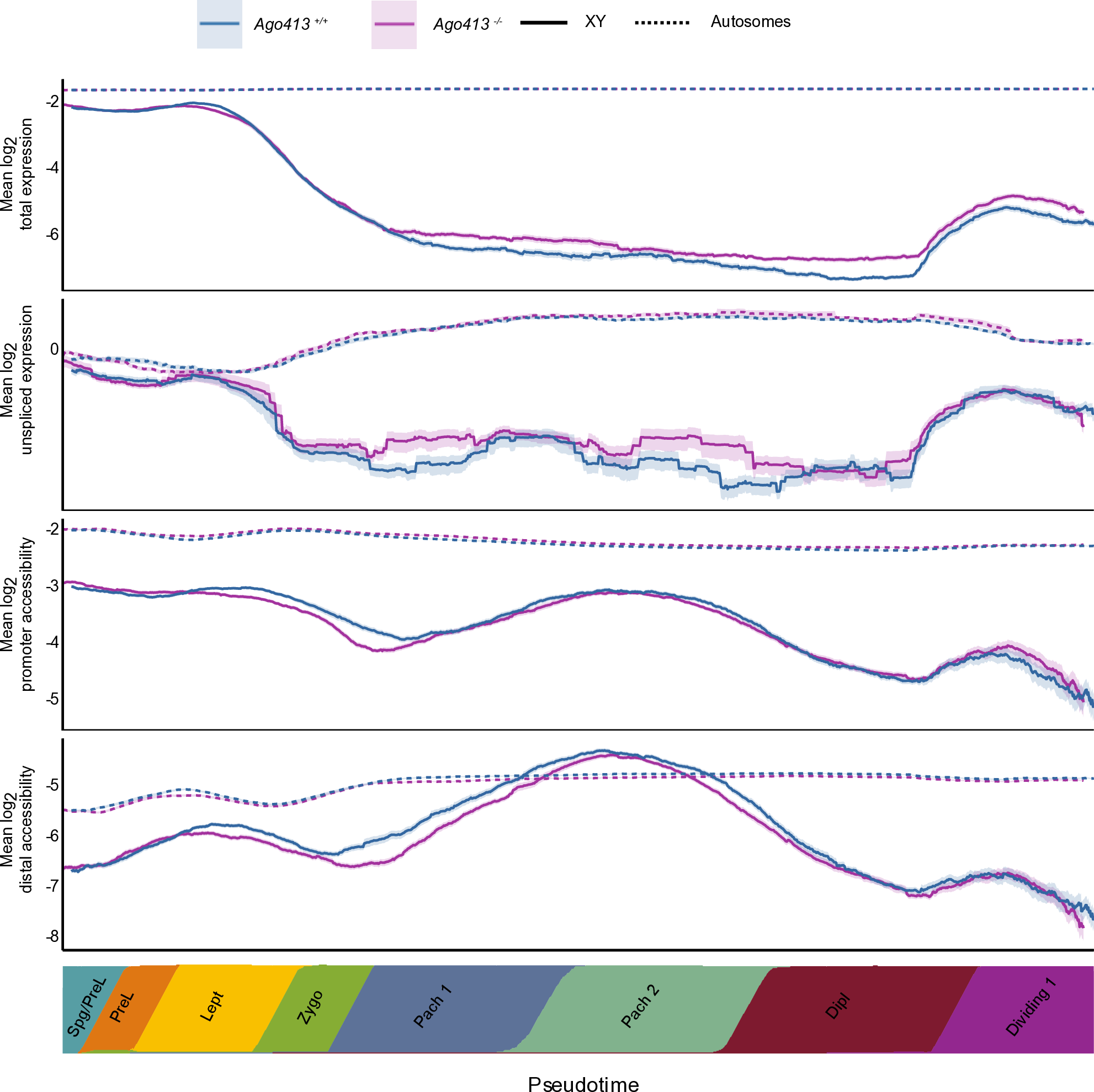

Supplement: S12 Fig — Bar plot of percentage of different cell types across pseudo time values for all genotypes. Pseudo time values were identified using the addSlingShotTrajectories function from ArchR on the combined UMAP and starting from the spermatogonia cluster. (TIF) [file pgen.1012217.s014.tif]
